# Exercise is associated with attenuated aging-related osteoporosis through TMAO alpha Klotho inflammasome signaling

**DOI:** 10.1016/j.isci.2026.117170

**Published:** 2026-08-11

**Authors:** Lihua Zhang, Xiaoyu Huang, Jiawei Zhang, Xiangyue Si, Hongchun Zuo, Qun Wang, Weijun Gong

**Affiliations:** 1 Department of Musculoskeletal Rehabilitation, Beijing Rehabilitation Hospital, Capital Medical University, Beijing 100144, China; 2 Beijing Rehabilitation Medicine Academy, Capital Medical University, Beijing 100144, China

**Keywords:** age-related osteoporosis, exercise, TMAO, α-Klotho-TXNIP/NLRP3 pathway, osteoblast senescence

## Abstract

Age-related osteoporosis is shaped by disrupted bone remodeling, metabolic stress, and inflammation. We combined an exploratory clinical comparison in adults aged 65 years and older with a D-galactose aging rat model and osteoblast-like cell experiments to examine exercise-associated changes in trimethylamine N-oxide (TMAO) and inflammasome signaling. Higher habitual activity in older adults was associated with higher hip T scores, lower serum and fecal TMAO, reduced IL-18 and IL-1β, and a turnover profile favoring bone formation. In aged rats, exercise lowered circulating and femoral marrow TMAO, preserved trabecular architecture, improved maximal load, and restrained TXNIP NOD-like receptor family pyrin domain-containing 3 (NLRP3) signaling while maintaining alpha Klotho. In osteoblast-like cells, TMAO promoted senescence and inflammasome assembly, whereas pathway modulation reduced these effects. These data support a gut bone inflammatory framework for exercise-associated skeletal protection in aging.

## Highlights

• Higher habitual activity was associated with higher hip BMD and lower TMAO
• Exercise attenuated TMAO-associated skeletal changes in aged rats
• TMAO exposure was associated with osteoblast senescence and inflammasome activation
• Higher α-Klotho was linked to less TXNIP-dependent inflammasome assembly

## Graphical abstract

**Figure:**
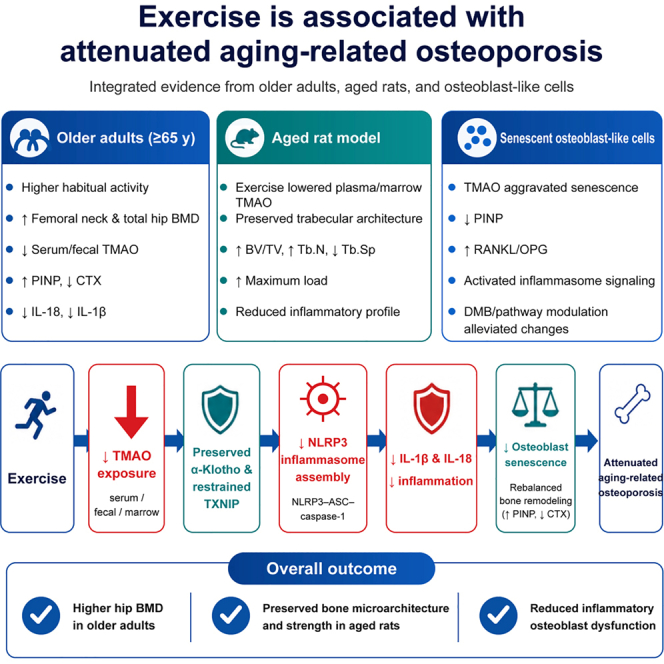

## Teaser

Health sciences; Physiology; Molecular biology; Immunology

## Introduction

Osteoporosis is characterized by trabecular deterioration and loss of bone mass.^1^^,^^2^ Its prevalence approaches 21.7% in adults aged 50–85 years, resulting in over 8.9 million fractures annually and constituting a major public health burden in aging societies.^3^^,^^4^^,^^5^ The core pathology is an imbalance between osteoblast and osteoclast activity, driven by hormonal changes, low-grade inflammation, oxidative stress, and abnormalities of the bone marrow microenvironment.^6^ Although antiresorptive and anabolic drugs can slow disease progression, safe upstream interventions targeting metabolic-inflammatory drivers of skeletal aging remain needed.^7^

Regular exercise improves bone quantity and strength, yet the molecular pathways by which it acts in skeletal aging remain insufficiently defined.^8^^,^^9^ Trimethylamine N-oxide (TMAO) is generated from trimethylamine produced by gut microbial metabolism of dietary choline, phosphatidylcholine, and L-carnitine. Elevated TMAO has been linked to chronic low-grade inflammation, oxidative stress, and multisystem aging.^10^ Mechanistically, TMAO reportedly suppresses osteogenic differentiation, enhances osteoclast activity, and thereby promotes unbalanced remodeling and bone loss.^11^^,^^12^ TMAO may also intensify inflammation by activating the NOD-like receptor family pyrin domain-containing 3 (NLRP3) inflammasome, a cytosolic multiprotein complex composed of NLRP3, the adaptor apoptosis-associated speck-like protein containing a CARD (ASC), and caspase-1.^13^ Activated NLRP3 promotes maturation and release of interleukin-1β (IL-1β) and interleukin-18 (IL-18), amplifying sterile inflammation and impairing osteoblast function.^14^ However, whether exercise-related modulation of TMAO is linked to osteoblast inflammasome signaling during skeletal aging remains unclear.

The α-Klotho-TXNIP/NLRP3 signaling axis represents a critical nexus regulating oxidative stress and inflammation. α-Klotho functions as an anti-aging protein with antioxidant properties that restrain pathological signaling.^15^ Conversely, TXNIP acts as a stress-responsive mediator that facilitates NLRP3 assembly and activation when protective regulation fails. We therefore hypothesized that exercise attenuates aging-related bone loss, at least in part, by reducing TMAO-associated stress and preserving α-Klotho-dependent restraint of the TXNIP/NLRP3 axis.

To test this hypothesis, we employed a translational design integrating a clinical cross-sectional study, a D-galactose-induced aging rat model, and osteoblast culture experiments. The clinical component evaluated associations between habitual exercise, serum TMAO, inflammatory cytokines (IL-18 and IL-1β), and bone mineral density (BMD) in adults aged ≥65 years. The animal component examined whether structured exercise attenuates the detrimental skeletal effects of exogenous TMAO. Finally, the cellular component interrogated the role of the α-Klotho-TXNIP/NLRP3 pathway in TMAO-induced osteoblast senescence using genetic modulation. This study aims to elucidate the molecular mechanisms of exercise-mediated osteoprotection and identify targetable upstream regulators for aging-related bone loss.

## Results

### Baseline characteristics of study participants

**Figure 1:**
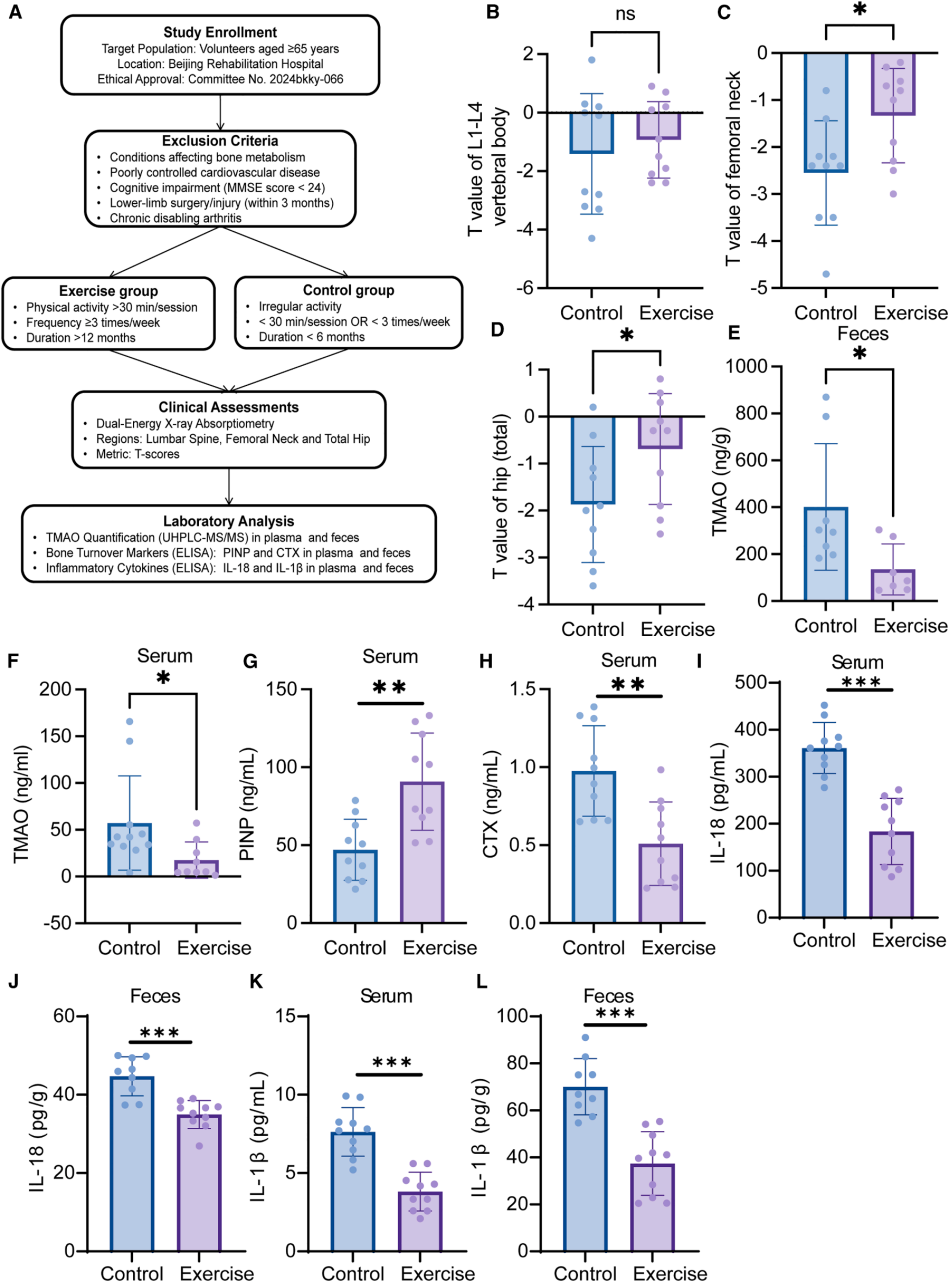
Higher habitual physical activity is associated with higher hip BMD, lower TMAO, a formation-favorable remodeling profile, and reduced inflammation in older adults (A) Flowchart of the clinical study design, illustrating participant enrollment, exclusion criteria, grouping according to habitual physical activity level, and outcome assessments. Participants were categorized into a higher activity group and a lower activity group based on self-reported habitual physical activity. (B–D) Comparison of bone mineral density T-scores at the (B) lumbar spine (L1–L4), (C) femoral neck, and (D) total hip. (E and F) TMAO concentrations measured in (E) feces and (F) serum. (G and H) Serum bone turnover markers showing (G) PINP and (H) CTX levels. (I–L) Inflammatory cytokine levels, including (I) serum IL-18, (J) fecal IL-18, (K) serum IL-1β, and (L) fecal IL-1β. Data are presented as mean ± SD; dots represent individual participants. *n* = 10 participants per group. For (B)–(L), between-group comparisons were performed using an unpaired two-tailed Student’s *t* test for normally distributed data or the Mann-Whitney U test otherwise, as specified in the STAR Methods. No post hoc test was applied for two-group comparisons. *p* < 0.05; ns, not significant. Abbreviations: BMD, bone mineral density; CTX, C-terminal telopeptide of type I collagen; PINP, procollagen type I N-terminal propeptide; TMAO, trimethylamine N-oxide.

A total of 20 older adults were included in the final analysis (*n* = 10 per group), as detailed in the study flow diagram (Figure 1A). The demographic and clinical characteristics of the study population are summarized in Table 1. No statistically significant differences were observed between groups regarding age, gender distribution, BMI, residence, or education level (all *p* > 0.05). This baseline comparability minimizes potential confounding effects on bone health outcomes.

**Table 1:** Baseline characteristics of the study participants

Characteristic	Higher activity (*n* = 10)	Lower activity (*n* = 10)	*p* value
Age, years	73.20 ± 9.75	71.40 ± 7.06	0.6425
Sex, *n* (%)	–	–	>0.9999
Male	3 (30)	3 (30)	–
Female	7 (70)	7 (70)	–
BMI, kg/m^2^	23.85 ± 3.70	24.27 ± 4.18	0.8129
Residence, *n* (%)	–	–	>0.9999
Rural	7 (70)	8 (80)	–
Urban	3 (30)	2 (20)	–
Education level, *n* (%)	–	–	>0.9999
Middle school or below	7 (70)	8 (80)	–
High school or above	3 (30)	2 (20)	–

Data are presented as mean ± SD or *n* (%). *p* values were calculated using an independent-samples *t* test for continuous variables and Fisher’s exact test for categorical variables.

### Higher habitual physical activity is associated with lower TMAO and inflammatory cytokines and a turnover profile favoring bone formation

Among participants aged 65 years or older, lumbar spine L1–L4 T-scores did not differ between groups stratified by self-reported habitual activity level (Figure 1B). Compared with the lower activity group, the higher activity group had significantly higher femoral neck and total hip T-scores (Figures 1C and 1D, *p* < 0.05). Metabolic and remodeling indices showed a concordant pattern: fecal and serum TMAO were lower in the higher activity group (Figures 1E and 1F, *p* < 0.05); the bone formation marker PINP was higher, whereas the bone resorption marker CTX was lower (Figures 1G and 1H, *p* < 0.05). Concomitantly, inflammation-related indices showed improvements consistent with the aforementioned trends; serum and fecal IL-18 and IL-1β levels in the higher activity group were significantly lower than those in the lower activity group (Figures 1I–1L, all *p* < 0.05). Together, these findings suggest that higher habitual activity levels were associated with a more favorable bone density phenotype, lower TMAO exposure, a shift toward bone formation, and attenuated systemic and gut-associated inflammation.

### Exercise mitigates TMAO-exacerbated bone fragility and microarchitectural deterioration in aged rats

**Figure 2:**
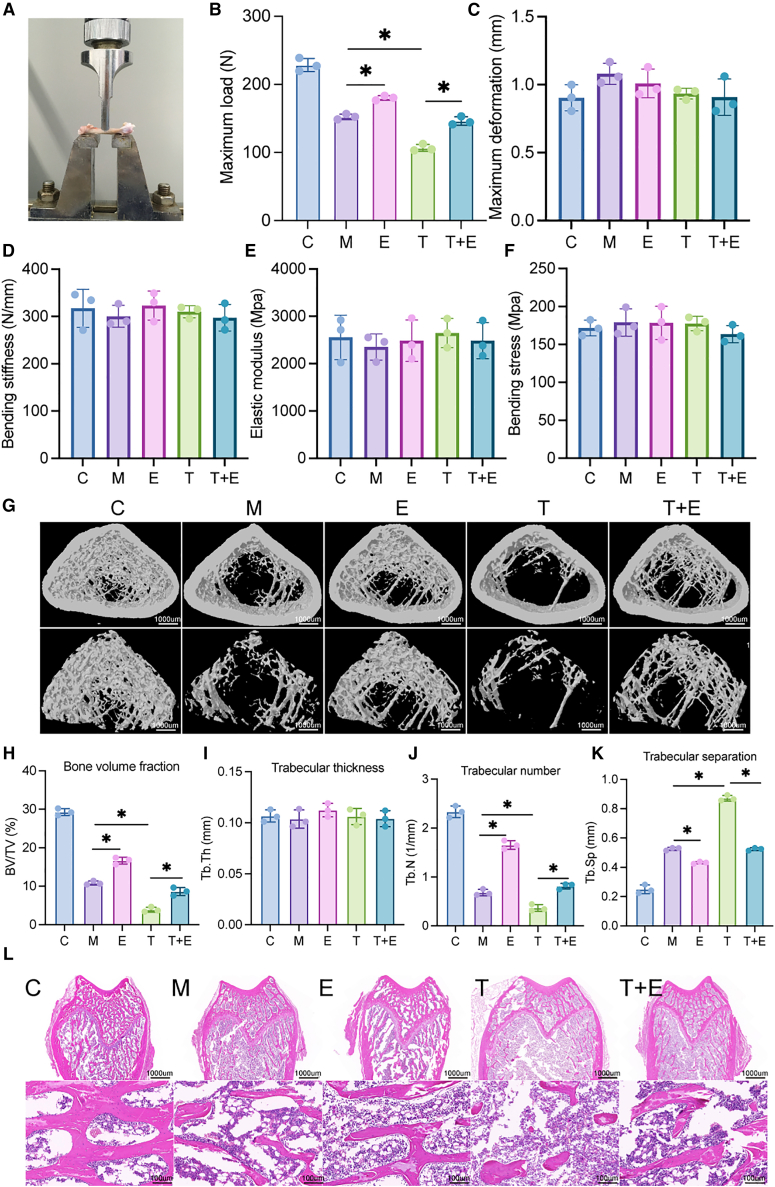
Exercise mitigates TMAO-induced loss of bone strength and mass in aged rats (A) Representative photograph of the three-point bending setup used for femoral biomechanical testing. (B–F) Mechanical properties of femora from control (C), model (M), exercise (E), TMAO (T), and TMAO plus exercise (T + E) groups, including (B) maximum load, (C) maximum deformation, (D) bending stiffness, (E) elastic modulus, and (F) bending stress. TMAO reduced maximum load compared with the model group, whereas exercise improved load-bearing capacity and partially restored maximum load in TMAO-exposed rats. No significant differences were observed in maximum deformation, bending stiffness, elastic modulus, or bending stress among groups. (G) Representative micro-CT reconstructions of the distal femoral metaphysis showing trabecular architecture across groups. Scale bars, 1,000 μm. (H–K) Quantitative micro-CT parameters, including (H) bone volume fraction (BV/TV), (I) trabecular thickness (Tb.Th), (J) trabecular number (Tb.N), and (K) trabecular separation (Tb.Sp). Exercise preserved BV/TV and Tb.N and reduced Tb.Sp, whereas TMAO aggravated trabecular deterioration. (L) Representative H&E staining of the distal femoral metaphysis. The upper part of the images show low-magnification images, and the lower part of the images show high-magnification trabecular regions. Scale bars, 1,000 μm in upper images and 100 μm in lower images. Data are presented as mean ± SD; dots represent individual animals. *n* = 3 rats per group. Statistical comparisons were performed using one-way ANOVA followed by Tukey’s multiple-comparisons post hoc test. ∗*p* < 0.05; ns, not significant. Group labels: C, control; M, model; E, exercise; T, TMAO; T + E, TMAO plus exercise.

We assessed the impact of exercise and TMAO on femoral bone quality using biomechanical testing, micro-computed tomography (CT), and histology (Figure 2A). Biomechanically, the aging model group exhibited compromised bone strength. Exercise training significantly enhanced the maximum load compared with the model group, whereas TMAO administration further reduced it. Notably, the combination of exercise and TMAO resulted in a partially restored maximum load compared with TMAO alone (Figure 2B; *p* < 0.05). No significant differences were observed in maximum deformation, bending stiffness, elastic modulus, or bending stress among groups (Figures 2C–2F). These findings indicate that the improvement in whole-bone strength with exercise was selective and was not accompanied by detectable changes in intrinsic material-related mechanical parameters.

Regarding microarchitecture, micro-CT imaging visualized marked trabecular bone loss in the model and TMAO groups (Figure 2G). Quantitative analysis confirmed that TMAO treatment significantly decreased bone volume fraction (BV/TV) and trabecular number (Tb.N) while increasing trabecular separation (Tb.Sp) relative to the model group (Figures 2H, 2J, and 2K; *p* < 0.05). Exercise, however, effectively attenuated these deleterious effects, maintaining BV/TV and Tb.N at levels comparable to or higher than the model group. Trabecular thickness (Tb.Th) showed no statistically significant variation (Figure 2I). Consistently, histological analysis (H&E staining) showed that femurs from the model and TMAO groups featured expanded marrow spaces and discontinuous trabeculae. In contrast, exercise intervention preserved trabecular integrity and connectivity (Figure 2L). Together, these results suggest that exercise primarily improved bone strength by preserving trabecular bone mass and microarchitecture, particularly BV/TV and Tb.N, rather than by altering intrinsic material properties.

### Exercise attenuates TMAO-induced systemic inflammation and rebalances bone turnover

**Figure 3:**
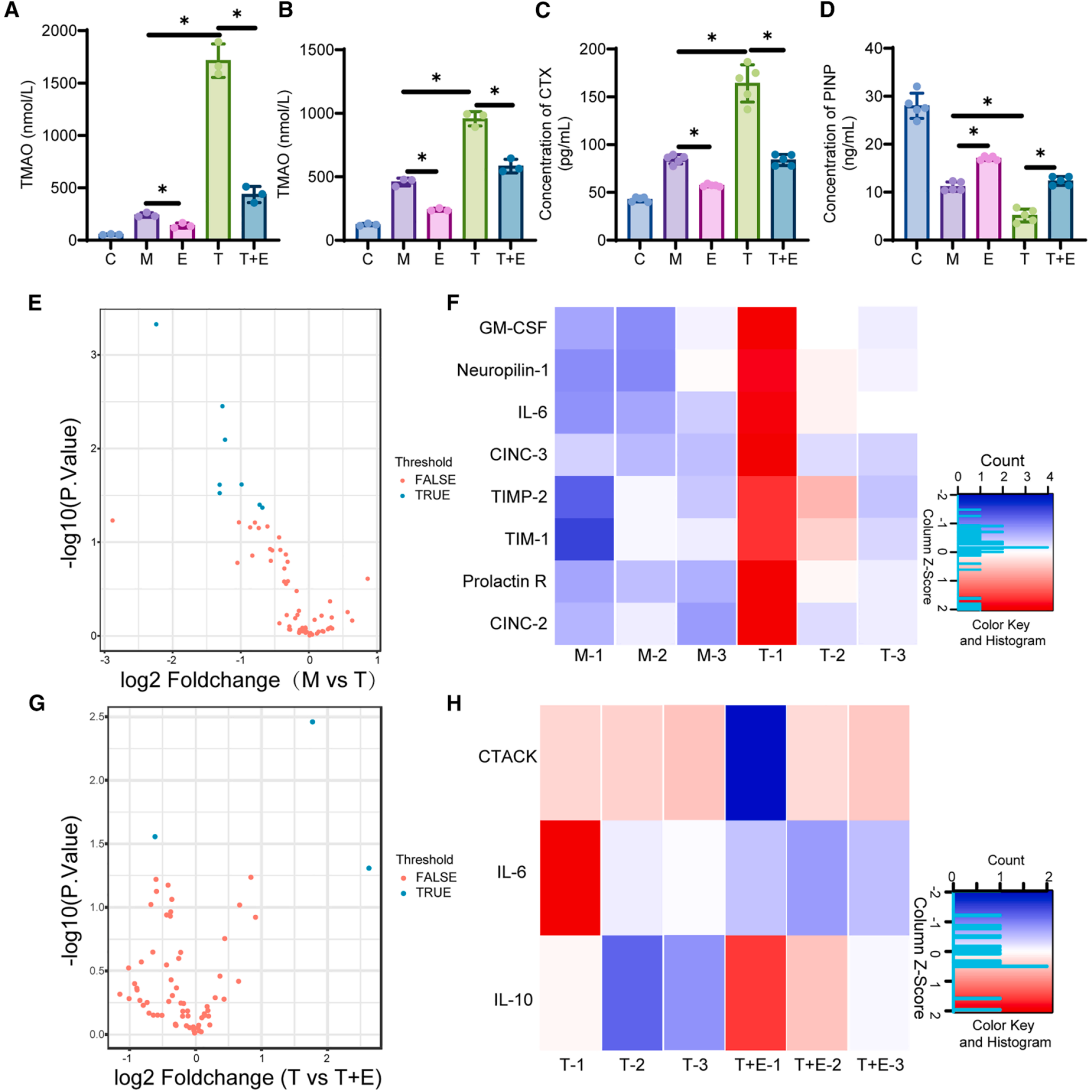
Exercise suppresses TMAO-induced bone resorption and systemic inflammation in aged rats (A and B) (A) Femoral marrow TMAO (ng/g) and (B) plasma TMAO (nmol/L) quantified by ultra‑high‑performance liquid chromatography‑mass spectrometry (UHPLC-MS)/MS. Exercise lowered TMAO relative to the model (E < M), whereas TMAO loading increased TMAO (T > M) and was partially rescued by exercise (T + E < T). (C) Serum CTX (pg/mL) was elevated by TMAO and reduced by exercise, consistent with attenuation of bone resorption. (D) Serum PINP (ng/mL) showed the opposite pattern—elevated with exercise, reduced by TMAO, and restored in T + E. (E) Volcano plot of differentially expressed circulating cytokines between model (M) and TMAO (T) groups; significantly altered proteins are highlighted. (F) Heatmap of selected cytokines across individual animals (M-1–M-3, T-1–T-3) showing higher IL-6, CINC-3, GM-CSF, and related mediators in T. (G) Volcano plot comparing T vs. T + E demonstrates cytokine shifts with exercise. (H) Heatmap illustrates a reduction in the pro-inflammatory cytokine IL-6 and an increase in the anti-inflammatory cytokine IL-10 in T + E relative to T. Bars indicate mean ± SD; dots are individual animals (*n* per group in STAR Methods). *n* = 3 rats per group for (A)–(D). Statistical comparisons for (A)–(D) were performed using one-way ANOVA followed by Tukey’s multiple-comparisons post hoc test. For cytokine antibody array analyses in (E)–(H), *n* = 3 rats per group; normalized signal intensities were used for two-group screening of differentially expressed cytokines, and heatmaps display normalized relative intensities. ∗*p* < 0.05; ns, not significant. Group labels: C, control; M, model; E, exercise; T, TMAO; T + E, TMAO plus exercise.

To determine the impact of exercise on TMAO accumulation and bone metabolism, we analyzed biomarkers in serum and marrow. Exercise training significantly lowered TMAO concentrations in both femoral marrow and plasma compared with the non-exercised aging model. Moreover, in the TMAO plus exercise group, exercise effectively counteracted the exogenous TMAO load (Figures 3A and 3B; *p* < 0.05). Analysis of bone turnover markers indicated a protective effect of exercise against TMAO-induced imbalance. The bone resorption marker CTX was significantly elevated in the TMAO group but was downregulated by concurrent exercise (Figure 3C). Conversely, the bone formation marker PINP, which was suppressed by TMAO, showed a significant recovery in the combined intervention group (Figure 3D). Inflammatory profiling via antibody arrays revealed distinct molecular signatures. Volcano plots identified significant differentially expressed proteins (Figures 3E and 3G). Heatmap visualization confirmed that TMAO administration elicited a robust pro-inflammatory response, characterized by the upregulation of IL-6, CINC-3, and GM-CSF relative to the model group (Figure 3F). Importantly, exercise treatment in TMAO-exposed rats (Figure 3H) reversed this profile by specifically suppressing pro-inflammatory cytokines such as IL-6 while simultaneously upregulating the anti-inflammatory cytokine IL-10. These findings suggest that exercise exerts osteoprotection by dampening TMAO-driven inflammation and fostering an anti-inflammatory microenvironment.

### TMAO exacerbates D-galactose-induced osteoblast senescence and suppresses osteogenesis

**Figure 4:**
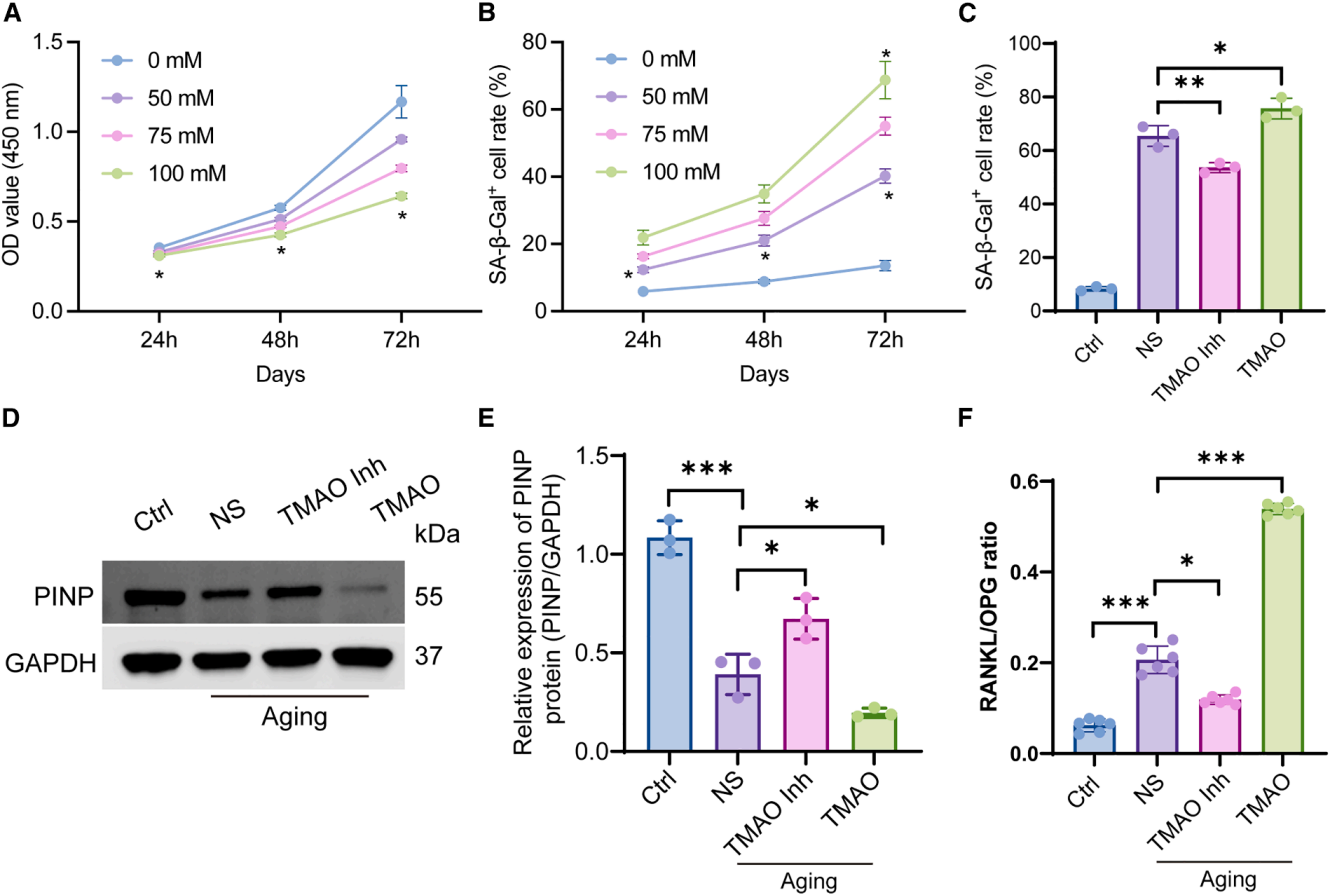
TMAO accelerates osteoblast senescence and suppresses osteogenic function *in vitro* (A and B) Optimization of the senescence model in rat osteoblasts using D-galactose (0, 50, 75, and 100 mM for 24–72 h). Cell viability (CCK-8, OD450) declined and SA-β-gal-positive cells increased in a dose- and time-dependent manner; 100 mM for 72 h was chosen for subsequent assays. (C) Senescence burden (SA-β-gal-positive rate) in non-aged control (Ctrl), D-gal-aged + vehicle (NS), D-gal-aged + TMAO (TMAO), and D-gal-aged + DMB treatment (TMAO Inh) groups. TMAO treatment further increased senescence positivity compared to the NS group, whereas DMB treatment mitigated this effect. (D) Representative immunoblots for the osteogenic marker PINP with GAPDH as the loading control. (E) Densitometric quantification (relative expression of PINP/GAPDH) shows that TMAO exposure suppresses PINP expression, while DMB treatment partially restores it. (F) ELISA-derived RANKL/OPG ratio in conditioned supernatants indicates that TMAO shifted osteoblast-derived signaling toward a more pro-osteoclastogenic state, whereas DMB treatment reduced this shift. Data are presented as mean ± SD. For (A) and (B), *n* = 3 independent experiments per concentration/time condition, and statistical comparisons were performed using one-way ANOVA followed by Tukey’s multiple-comparisons test. For (C) and (F), *n* = 3 independent biological replicates per group, and comparisons were performed using one-way ANOVA followed by Tukey’s multiple-comparisons post hoc test. For (E), *n* = 3 independent western blot experiments and comparisons were performed using one-way ANOVA followed by Tukey’s multiple-comparisons post hoc test. ∗*p* < 0.05; ∗∗*p* < 0.01; ∗∗∗*p* < 0.001; ns, not significant. Abbreviations: DMB, 3,3-dimethyl-1-butanol; PINP, procollagen type I N-terminal propeptide; RANKL, receptor activator of nuclear factor-kappa B ligand; OPG, osteoprotegerin.

To establish an optimal *in vitro* senescence model, ROS17/2.8 cells were exposed to increasing concentrations of D-galactose over various durations. CCK-8 assays revealed that higher D-galactose concentrations progressively reduced cell viability (Figure 4A), while SA-β-gal staining showed a dose- and time-dependent increase in the proportion of senescent cells (Figure 4B). Based on these optimizations, treatment with 100 mM D-galactose for 72 h was selected for subsequent experiments. Under these validated senescence conditions, exogenous TMAO significantly exacerbated cellular senescence, as evidenced by a further increase in SA-β-gal positivity compared with the senescence control group. Conversely, co-treatment with the 3,3-dimethyl-1-butanol (DMB) significantly attenuated this effect (Figure 4C). Regarding osteogenic function, western blot analysis demonstrated that protein expression of the bone formation marker PINP was markedly suppressed by TMAO but was partially rescued by DMB treatment (Figures 4D and 4E). To further evaluate whether these osteoblast changes might indirectly influence osteoclastogenic signaling, we assessed the receptor activator of nuclear factor-κB ligand (RANKL)/osteoprotegerin (OPG) ratio in conditioned supernatants. TMAO significantly increased the RANKL/OPG ratio compared with the senescent control group, whereas DMB treatment reduced this increase (Figure 4F), suggesting a shift toward a more pro-osteoclastogenic paracrine milieu. Collectively, these findings indicate that TMAO aggravates senescence phenotypes, compromises the osteogenic capacity of osteoblast-like cells, and may indirectly favor bone resorption by altering osteoblast-derived osteoclast-regulatory signaling.

### TMAO activates the α-Klotho-TXNIP/NLRP3 signaling pathway, promotes NLRP3 inflammasome assembly, and increases downstream cytokine release

**Figure 5:**
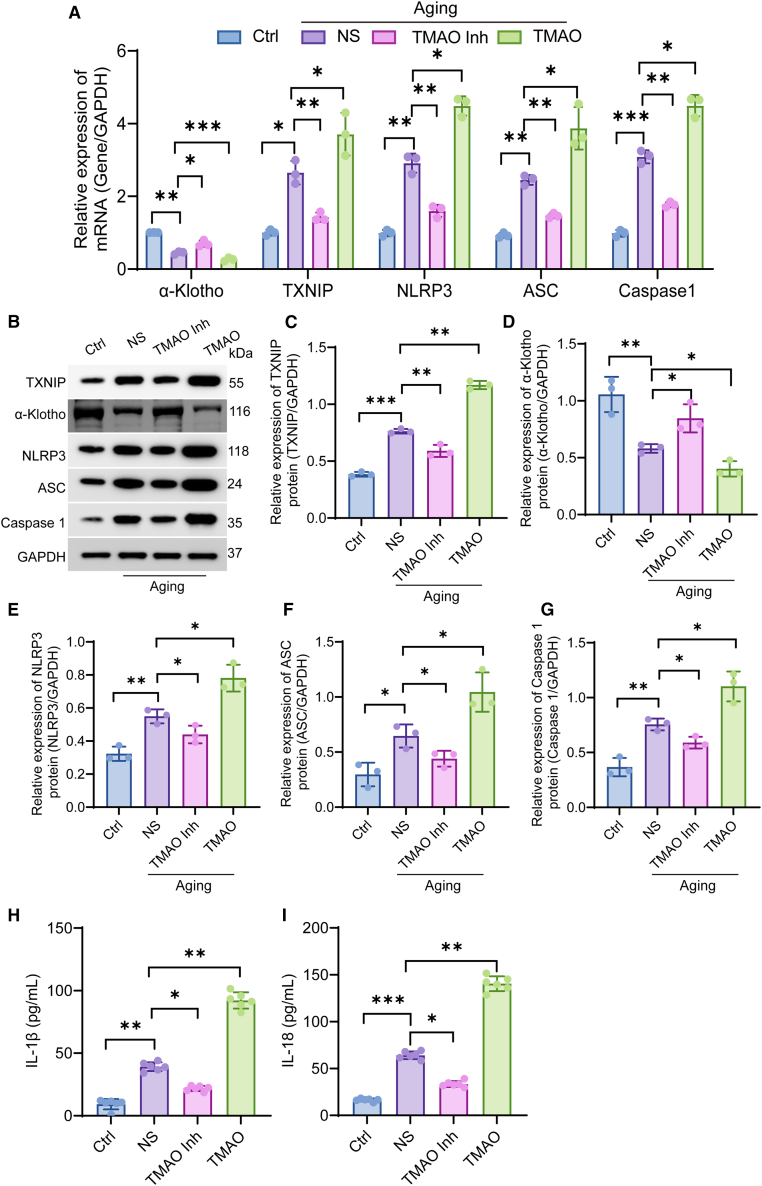
TMAO activates the TXNIP/α-Klotho/NLRP3 axis and upregulates inflammasome components in aged osteoblasts, which is reversed by DMB treatment Rat osteoblasts were subjected to D-gal-induced aging and treated with vehicle (NS), DMB (TMAO Inh), or TMAO, with a non-aged control (Ctrl). (A) RT-qPCR analysis of α-Klotho, TXNIP, NLRP3, ASC, and caspase-1 mRNA levels normalized to GAPDH shows that TMAO increases TXNIP, NLRP3, ASC, and caspase-1 while decreasing α-Klotho; TMAO inhibition mitigates these changes. (B) Representative immunoblots for the indicated proteins with GAPDH as the loading control. (C–G) Densitometric quantification of TXNIP, α-Klotho, NLRP3, ASC, and caspase-1 protein abundance relative to GAPDH confirms the transcriptional trends. (H and I) ELISA of culture supernatants shows that TMAO increased the secretion of (H) IL-1β and (I) IL-18, whereas DMB treatment attenuated these increases. Bars represent mean ± SD. For (A), *n* = 3 independent biological replicates per group. For (C)–(G), *n* = 3 independent western blot experiments. For (H) and (I), *n* = 3 independent biological replicates per group. Statistical comparisons were performed using one-way ANOVA followed by Tukey’s multiple-comparisons post hoc test. ∗*p* < 0.05; ∗∗*p* < 0.01; ∗∗∗*p* < 0.001; ns, not significant. Abbreviations: DMB, 3,3-dimethyl-1-butanol; TXNIP, thioredoxin-interacting protein; ASC, apoptosis-associated speck-like protein containing a CARD.

To elucidate the molecular mechanism driving TMAO-induced bone impairment, we investigated the α-Klotho-TXNIP/NLRP3 axis using the established osteoblast senescence model. RT-qPCR and western blot analyses consistently demonstrated that TMAO treatment significantly upregulated the mRNA and protein expression of TXNIP and the inflammasome components NLRP3, ASC, and caspase-1, while concurrently downregulating α-Klotho (Figures 5A–5G). DMB treatment attenuated these TMAO-induced molecular perturbations. To validate these findings *in vivo*, we analyzed femoral bone tissue from aged rats. *In vivo* RT-qPCR analysis showed that TMAO exposure increased the transcript levels of TXNIP, NLRP3, ASC, and caspase-1, while suppressing α-Klotho expression (Figures S1A–S1E), thereby providing *in vivo* transcriptional support for the mechanistic changes observed *in vitro*. To directly assess the downstream biological consequences of inflammasome activation, we quantified IL-1β and IL-18 in culture supernatants by ELISA. Compared with the senescent control condition, TMAO exposure markedly increased the release of both IL-1β and IL-18, whereas DMB treatment attenuated these increases (Figures 5H and 5I). Together, these Figure 5 data indicate that TMAO suppresses α-Klotho, activates the TXNIP/NLRP3 axis, and increases downstream inflammatory cytokine release in senescent osteoblast-like cells.

**Figure 6:**
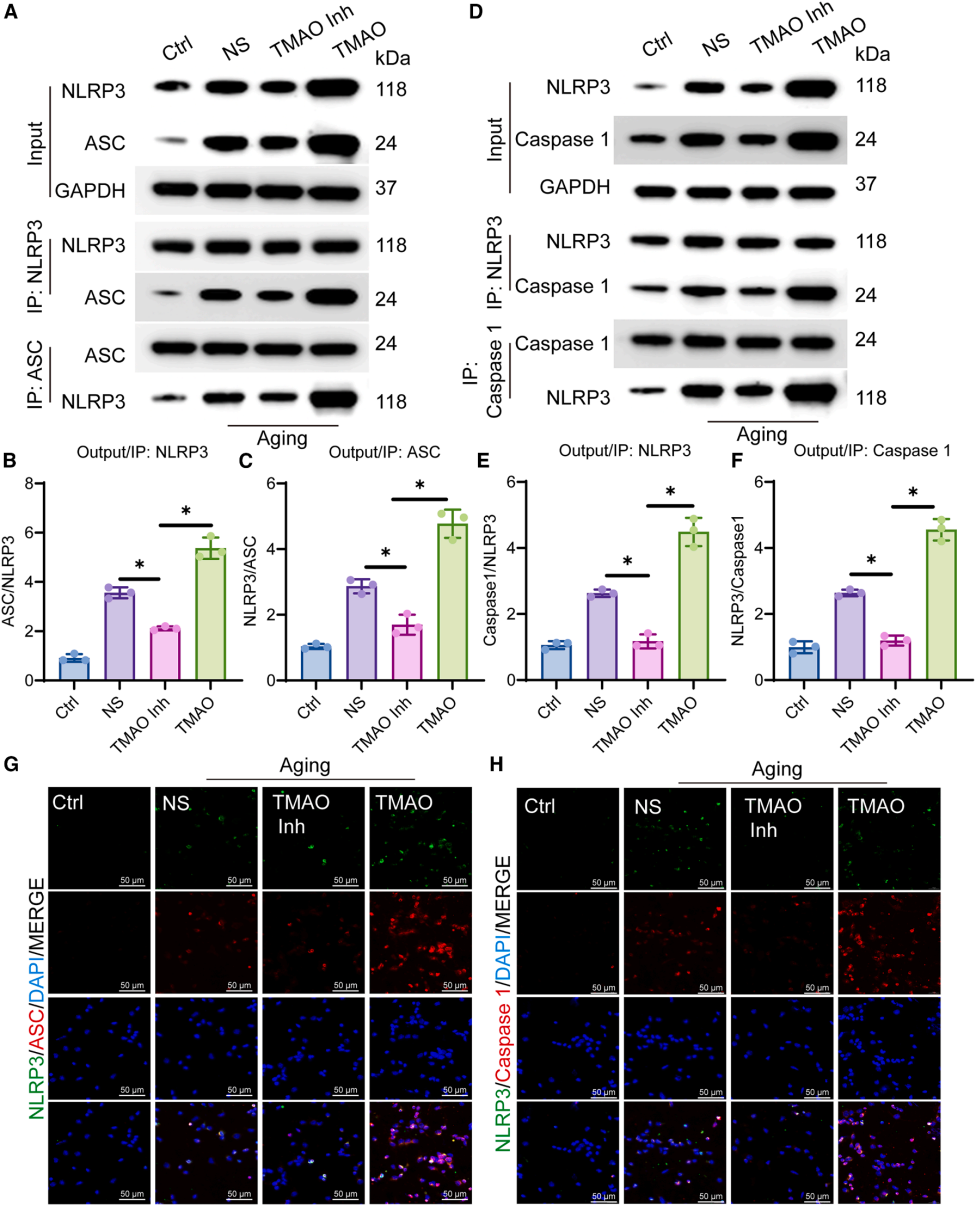
TMAO promotes NLRP3 inflammasome assembly and colocalization of inflammasome components in senescent osteoblast-like cells Rat osteoblast-like cells were subjected to D-galactose-induced aging and treated with vehicle (NS), DMB (TMAO Inh), or TMAO, with non-aged cells used as the control (Ctrl). (A) Co-immunoprecipitation (coIP) with anti-NLRP3 or anti-ASC antibodies showing the interaction between NLRP3 and ASC. Representative input and immunoprecipitated protein blots are shown. Molecular weight markers are indicated in kDa. (B and C) Densitometric quantification of (B) ASC/NLRP3 in NLRP3 immunoprecipitates and (C) NLRP3/ASC in ASC immunoprecipitates. (D) coIP with anti-NLRP3 or anti-caspase-1 antibodies showing the interaction between NLRP3 and caspase-1. Representative input and immunoprecipitated protein blots are shown. Molecular weight markers are indicated in kDa. (E and F) Densitometric quantification of (E) caspase-1/NLRP3 in NLRP3 immunoprecipitates and (F) NLRP3/caspase-1 in caspase-1 immunoprecipitates. TMAO increased NLRP3 inflammasome complex formation, whereas DMB treatment reduced these interactions. (G) Representative double immunofluorescence images showing NLRP3, ASC, DAPI nuclear staining, and merged NLRP3/ASC signals. (H) Representative double immunofluorescence images showing NLRP3, caspase-1, DAPI nuclear staining, and merged NLRP3/caspase-1 signals. TMAO increased punctate colocalization signals, whereas DMB attenuated this pattern. Scale bars, 50 μm. Data are presented as mean ± SD. For (B), (C), (E), and (F), *n* = 3 independent coIP experiments per group. Statistical comparisons were performed using one-way ANOVA followed by Tukey’s multiple-comparisons post hoc test. ∗*p* < 0.05; ∗∗*p* < 0.01; ns, not significant. Unedited representative immunofluorescence images corresponding to (G) and (H) are provided in the supplemental information. Abbreviations: ASC, apoptosis-associated speck-like protein containing a CARD; coIP, co-immunoprecipitation; DAPI, 4′,6-diamidino-2-phenylindole; DMB, 3,3-dimethyl-1-butanol; IP, immunoprecipitation; NLRP3, NOD-like receptor family pyrin domain-containing 3; TMAO, trimethylamine N-oxide.

We next examined whether these molecular changes were accompanied by NLRP3 inflammasome assembly. Co-immunoprecipitation analysis showed that TMAO enhanced NLRP3 inflammasome complex formation, as evidenced by increased recruitment of ASC to NLRP3 and elevated binding of caspase-1 to NLRP3. Conversely, DMB treatment weakened these interactions, suggesting reduced inflammasome assembly (Figures 6A–6F).

To complement these biochemical interaction data with spatial evidence, we performed double immunofluorescence staining for NLRP3/ASC and NLRP3/caspase-1. Control cells showed weak and largely diffuse staining with minimal overlap signals. D-galactose-induced senescence was accompanied by partial colocalization of NLRP3 with ASC and caspase-1. TMAO treatment markedly increased punctate merged signals in both NLRP3/ASC and NLRP3/caspase-1 staining, indicating enhanced spatial recruitment of ASC and caspase-1 to NLRP3, whereas DMB treatment attenuated these changes (Figures 6G and 6H).

Together, the co-immunoprecipitation and double-immunofluorescence results support that TMAO promotes the spatial and biochemical features of NLRP3 inflammasome assembly, rather than merely increasing the expression of inflammasome components.

### The α-Klotho-TXNIP/NLRP3 axis functionally regulates osteoblast senescence through reciprocal regulation and inflammasome assembly

**Figure 7:**
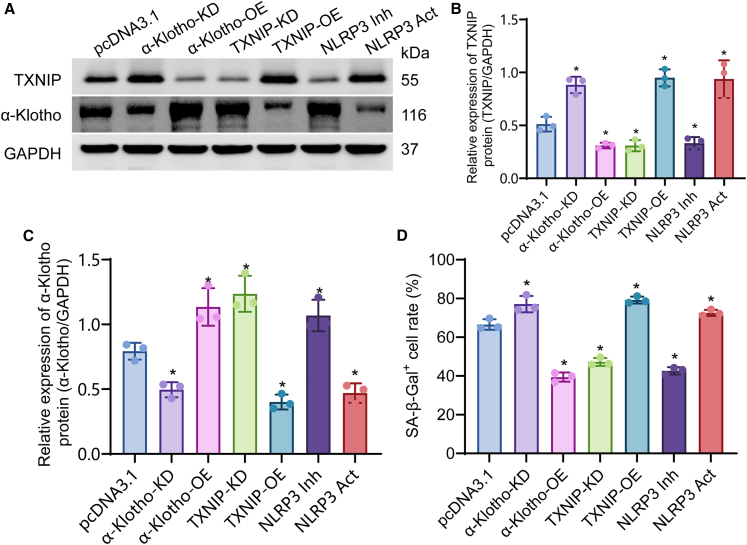
The TXNIP/α-Klotho/NLRP3 axis governs osteoblast senescence Rat osteoblasts were D-gal aged and subjected to genetic or pharmacologic perturbations: empty vector (pcDNA3.1), α-Klotho knockdown (α-Klotho-KD), α-Klotho overexpression (α-Klotho-OE), TXNIP knockdown (TXNIP-KD), TXNIP overexpression (TXNIP-OE), NLRP3 inhibitor (NLRP3-Inh; treated with 1 μM MCC950 for 24 h), or NLRP3 activator (NLRP3-Act; treated with 100 ng/mL LPS for 24 h). (A) Representative immunoblots for TXNIP and α-Klotho (GAPDH loading control). (B and C) Densitometry (protein/GAPDH) shows that α-Klotho-KD or NLRP3-Act elevates TXNIP, whereas TXNIP-KD or NLRP3-Inh lowers TXNIP (B); conversely, α-Klotho expression increases with TXNIP-KD or NLRP3-Inh and decreases with TXNIP-OE or NLRP3-Act (C). (D) Senescence burden quantified by SA-β-gal positivity demonstrates that TXNIP-OE and NLRP3-Act promote, while α-Klotho-OE, TXNIP-KD, and NLRP3-Inh attenuate, osteoblast senescence. Data are presented as mean ± SD. For (B) and (C), *n* = 3 independent western blot experiments per group. For (D), *n* = 3 independent biological replicates per group. Statistical comparisons were performed using one-way ANOVA followed by Tukey’s multiple-comparisons post hoc test. ∗*p* < 0.05; ∗∗*p* < 0.01; ∗∗∗*p* < 0.001; ns, not significant. Abbreviations: KD, knockdown; OE, overexpression; Inh, inhibitor; Act, activator.

**Figure 8:**
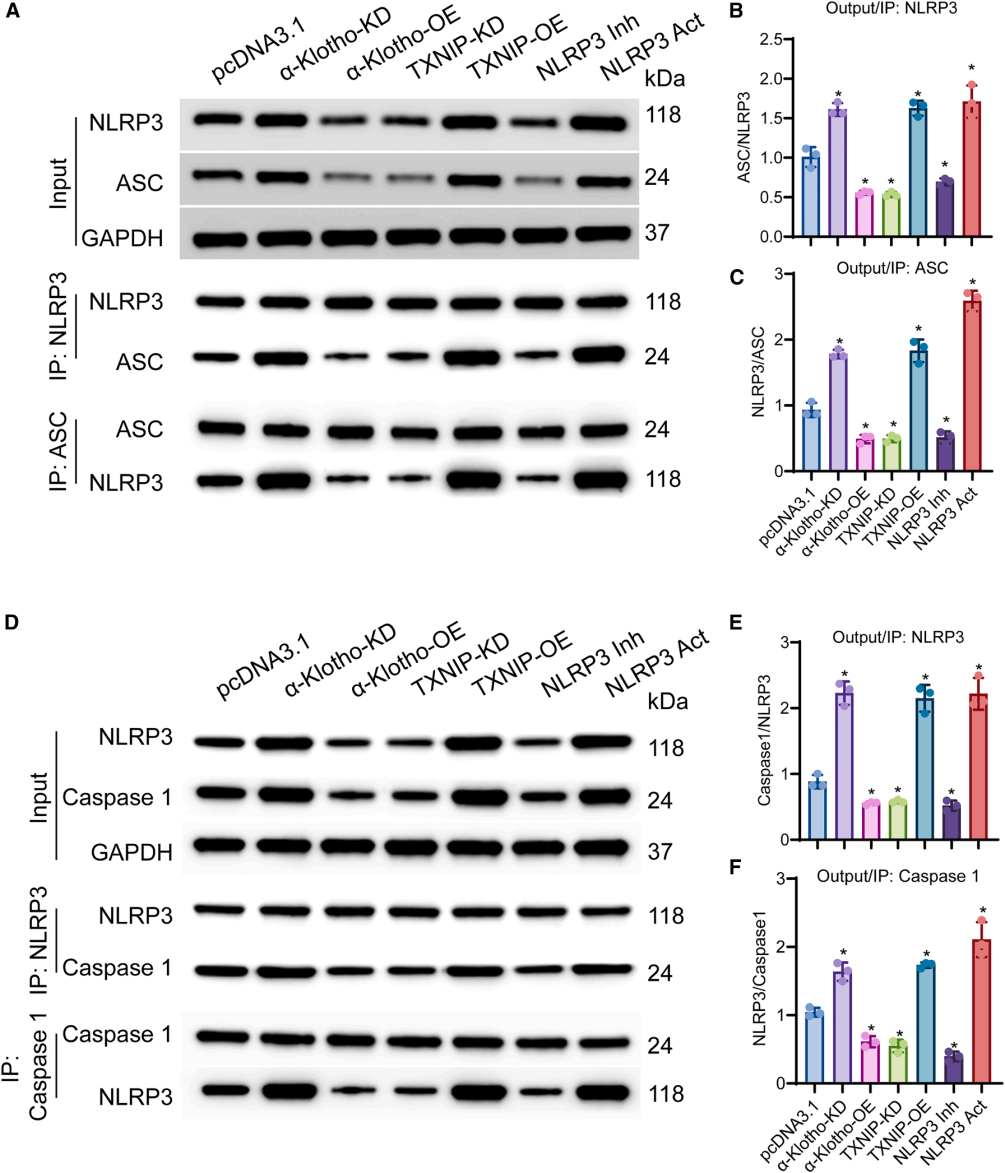
TXNIP promotes, whereas α-Klotho restrains, assembly of the NLRP3 inflammasome in osteoblasts Rat osteoblasts (D-gal aged) were subjected to the indicated perturbations: empty vector (pcDNA3.1), α-Klotho knockdown (α-Klotho-KD), α-Klotho overexpression (α-Klotho-OE), TXNIP knockdown (TXNIP-KD), TXNIP overexpression (TXNIP-OE), NLRP3 inhibitor (NLRP3-Inh; 1 μM MCC950), or NLRP3 activator (NLRP3-Act; 100 ng/mL LPS). (A) Co-immunoprecipitation (coIP) with anti-NLRP3 or anti-ASC shows altered NLRP3-ASC association across groups; inputs (top) document total protein (GAPDH loading control). (B and C) Densitometric quantification of ASC/NLRP3 in NLRP3 immunoprecipitates (B) and NLRP3/ASC in ASC immunoprecipitates (C). (D) coIP with anti-caspase-1 or anti-NLRP3 evaluates the NLRP3-caspase-1 interaction. (E and F) Quantification of caspase-1/NLRP3 in NLRP3 immunoprecipitates (E) and NLRP3/caspase-1 in caspase-1 immunoprecipitates (F). TXNIP-OE, α-Klotho-KD, and NLRP3-Act enhanced NLRP3 complex formation, whereas α-Klotho-OE, TXNIP-KD, and NLRP3-Inh reduced it. Data are presented as mean ± SD from *n* = 3 independent coIP experiments per group. Statistical comparisons for (B), (C), (E), and (F) were performed using one-way ANOVA followed by Tukey’s multiple-comparisons post hoc test. ∗*p* < 0.05; ∗∗*p* < 0.01; ns, not significant. Abbreviations: coIP, co-immunoprecipitation; KD, knockdown; OE, overexpression; Inh, inhibitor; Act, activator.

We first verified the efficacy of genetic manipulations *in vitro*. Western blotting confirmed that specific small interfering RNAs (siRNAs) effectively reduced while overexpression constructs significantly elevated the protein levels of α-Klotho and TXNIP, respectively (Figures S2A–S2D). Based on these validated models, we observed a bidirectional regulatory relationship between α-Klotho and the TXNIP/NLRP3 axis: loss of α-Klotho increased TXNIP expression and favored NLRP3 activation, whereas α-Klotho overexpression suppressed TXNIP and attenuated inflammasome assembly; conversely, TXNIP overexpression or NLRP3 activation reduced α-Klotho expression, while TXNIP knockdown or NLRP3 inhibition restored α-Klotho levels. Knockdown of α-Klotho significantly upregulated TXNIP protein levels, whereas its overexpression suppressed TXNIP. Consistently, pharmacological activation of NLRP3 using lipopolysaccharide (LPS) (100 ng/mL for 24 h) elevated TXNIP, while NLRP3 inhibition with MCC950 (1 μM for 24 h) markedly suppressed TXNIP protein levels (Figures 7A and 7B). Conversely, silencing TXNIP or inhibiting NLRP3 restored α-Klotho expression, whereas TXNIP overexpression or NLRP3 activation significantly suppressed it (Figures 7A and 7C). Functionally, SA-β-gal staining revealed that the senescence phenotype was tightly controlled by this axis: overexpression of α-Klotho or knockdown of TXNIP attenuated senescence, while α-Klotho silencing, TXNIP overexpression, or NLRP3 activation exacerbated the senescence burden (Figure 7D). Mechanistically, coIP assays (Figure 8) elucidated the assembly dynamics. Overexpression of TXNIP or activation of NLRP3 significantly promoted the recruitment of ASC and caspase-1 to NLRP3 (Figures 8A–8F). In contrast, α-Klotho overexpression or NLRP3 inhibition disrupted these interactions, thereby impeding inflammasome assembly. Collectively, these results indicate that α-Klotho antagonizes osteoblast senescence by disrupting the TXNIP-dependent assembly of the NLRP3 inflammasome.

## Discussion

This study identifies a TMAO-linked metabolic-inflammatory mechanism through which exercise may protect against aging-related bone loss. The main contribution of this study lies in connecting a gut-derived metabolite, osteoblast inflammasome activation, and bone remodeling within an integrated gut-bone framework. Across clinical, animal, and cellular levels, exercise-associated skeletal protection converged on lower TMAO exposure, reduced inflammatory output, preservation of trabecular microarchitecture, and restoration of a more formation-favorable remodeling profile. Mechanistically, our data support the α-Klotho-TXNIP/NLRP3 axis as a stress-responsive node linking TMAO exposure to osteoblast senescence, NLRP3-ASC-caspase-1 inflammasome assembly, IL-1β/IL-18 release, and a pro-osteoclastogenic RANKL/OPG shift.

Prior work has implicated TMAO in oxidative stress, nuclear factor-κB (NF-κB) activation, impaired osteogenic differentiation, and enhanced osteoclastogenesis.^16^^,^^17^^,^^18^ Our findings extend this concept by placing osteoblasts at the center of a TMAO-sensitive remodeling circuit. Osteoblasts not only execute bone formation but also regulate osteoclast differentiation through RANKL and OPG. Therefore, TMAO-induced osteoblast senescence may impair bone formation directly and, through an increased RANKL/OPG ratio, create a more pro-osteoclastogenic paracrine milieu. Consistent with this interpretation, the TMAO-induced increase in the osteoblast-derived RANKL/OPG ratio was attenuated by DMB, supporting a paracrine shift that may indirectly favor bone resorption rather than directly proving enhanced osteoclast differentiation. When interpreted together with the selective improvement in maximum load and preservation of BV/TV and Tb.N, these findings suggest that exercise primarily enhances whole-bone strength by maintaining trabecular bone mass and osteoblast-osteoclast coupling, rather than by measurably altering intrinsic material properties. Together, these observations support a contributory role for TMAO in the gut-blood-bone axis, while not establishing TMAO as an essential mediator of exercise-induced bone protection.

The reciprocal regulation between α-Klotho and TXNIP/NLRP3 can be interpreted as a self-amplifying inflammatory stress loop. Under TMAO-associated oxidative stress, reduced α-Klotho weakens antioxidant restraint, allowing TXNIP induction and NLRP3 inflammasome assembly.^19^ TXNIP then acts as a redox-sensitive adapter that facilitates NLRP3 recruitment of ASC and caspase-1, thereby promoting caspase-1 activation and downstream IL-1β/IL-18 maturation.^20^^,^^21^ In turn, activated TXNIP/NLRP3 signaling may further suppress α-Klotho expression and amplify inflammatory stress, thereby sustaining osteoblast senescence. Thus, loss of α-Klotho and activation of TXNIP/NLRP3 reinforce each other, converting transient metabolic stress into persistent inflammasome activation and impaired osteoblast function.^22^^,^^23^^,^^24^

An intriguing observation in our study is that pharmacologic inhibition of NLRP3 was accompanied by a reduction in TXNIP levels. Although TXNIP is canonically positioned upstream of NLRP3 as a docking adapter, our data suggest a bidirectional interplay. One plausible explanation is that blocking NLRP3 activation interrupts IL-1β/IL-18-driven autocrine and paracrine signaling, thereby attenuating NF-κB and other stress-responsive pathways that normally sustain TXNIP transcription. In addition, suppression of caspase-1-dependent pyroptosis and associated mitochondrial damage can secondarily reduce mitochondrial reactive oxygen species (ROS), a major driver of TXNIP induction and stabilization. Thus, NLRP3 inhibition not only prevents TXNIP from engaging NLRP3 but also feeds back to lower TXNIP expression by mitigating ROS and inflammatory signaling, effectively breaking the vicious α-Klotho-TXNIP/NLRP3 loop at multiple nodes.

Regarding the exercise-TMAO relationship, while acute high-intensity exercise can transiently spike TMAO, chronic training is known to remodel the microbiome, enriching butyrate producers and lowering basal TMAO.^25^^,^^26^^,^^27^ In our aging and bone-susceptible context, we observed steady-state reductions in TMAO alongside improved bone metabolism in both active adults and trained rats. These data support the view that durable adaptations in host-microbiome metabolism, rather than transient post-exercise fluctuations, underpin the skeletal benefits of exercise.

At the level of upstream signaling, exercise training elicits adaptive responses that promote cellular homeostasis. Specifically, exercise has been reported to increase circulating and tissue-resident α-Klotho, which restrains NLRP3 activation via antioxidant networks involving Nrf2 and FoxO, as well as calcium-phosphate regulatory pathways.^28^^,^^29^^,^^30^ By maintaining α-Klotho, exercise may buffer TMAO-induced oxidative stress and thereby limit excessive TXNIP induction and dissociation from thioredoxin. Notably, although our data support reciprocal regulation between α-Klotho and the TXNIP/NLRP3 axis, the precise molecular basis by which α-Klotho suppresses TXNIP expression was not fully resolved in the present study. Current evidence suggests that this effect is more likely mediated indirectly through redox control and antioxidant stress-response pathways than through a directly established TXNIP regulatory mechanism. Concurrently, exercise-induced activation of the AMPK/SIRT axis suppresses TXNIP transcription, accelerates its degradation, and reduces mitochondrial ROS, thereby dampening inflammasome priming.^31^^,^^32^^,^^33^ Together, these observations support a model in which α-Klotho and other exercise-responsive protective pathways converge to restrain ROS-driven TXNIP induction and downstream inflammasome activation. In our study, the observed shift in the IL-6/IL-10 ratio and the restoration of the PINP/CTX balance are consistent with such upstream protection and with re-alignment of the α-Klotho-TXNIP/NLRP3 axis and NLRP3-ASC-caspase-1 complex dynamics toward a less inflammatory, more bone-preserving state. Further work is needed to define the intermediate molecular steps linking α-Klotho to TXNIP regulation in osteoblasts. These findings reinforce the rationale for developing pharmacologic mimics of exercise signaling to combat age-related skeletal decline.

From an integrated gut-bone perspective, our findings expand the understanding of how gut-derived metabolites reprogram skeletal immunometabolism.^34^^,^^35^ While prior work has largely focused on short-chain fatty acids as beneficial regulators, evidence regarding TMAO as a distinct bone-toxic factor—and whether exercise can neutralize it—has been limited. Within a unified framework, our data position the α-Klotho-TXNIP/NLRP3 axis and the NLRP3-ASC-caspase-1 interaction network as a critical nexus connecting systemic metabolism, sterile inflammation, and bone remodeling. Exercise appears to break the “vicious cycle” of TMAO accumulation and α-Klotho loss, thereby restricting TXNIP-dependent NLRP3 activation and ASC/caspase-1 recruitment and correcting the imbalance between osteoblast and osteoclast activity. This protection likely propagates through crosstalk with marrow immune cells, potentially influencing downstream effectors such as the RANKL/OPG ratio and Wnt signaling,^36^^,^^37^ alongside potential synergistic inputs from muscle-derived myokines.^38^

At the same time, the present data should not be interpreted as proving that TMAO is an essential mediator of exercise-induced bone protection. Although exogenous TMAO loading aggravated skeletal and cellular phenotypes, and exercise partly reversed these alterations *in vivo*, the current design did not include microbiota-directed manipulation, TMA/TMAO synthesis inhibition, or TMAO depletion in exercised animals. Thus, our results support a functional contributory role for TMAO but do not establish a strict necessity model. In addition, no direct biochemical evidence was obtained to demonstrate direct binding of TMAO to α-Klotho or TXNIP, and the observed pathway changes should therefore be interpreted as functional associations rather than direct molecular interactions. Plasma TMAO and femoral marrow TMAO were included as complementary systemic and local exposure readouts, respectively, and were interpreted primarily on the basis of directional between-group changes rather than direct quantitative equivalence between the two compartments.

An important consideration in our study design was the deliberate focus on age-related rather than early-onset osteoporosis. Although young-adult or juvenile models may be valuable for investigating bone-intrinsic mechanisms under a more controlled biological background, our objective here was to examine the exercise-TMAO-α-Klotho-TXNIP/NLRP3 axis in the context of skeletal aging. In this setting, age-associated changes such as TMAO accumulation, reduced α-Klotho signaling, and osteoblast senescence were not treated as incidental background abnormalities but as integral components of the pathogenic milieu under investigation. Therefore, an aging model was considered more appropriate for addressing the specific metabolic-inflammatory mechanisms targeted in this study.

In conclusion, exercise in older adults is associated with higher hip bone mineral density and a remodeling pattern that favors formation. Mechanistically, regular exercise was associated with lower TMAO levels and reduced activation of the α-Klotho-TXNIP/NLRP3 axis, accompanied by attenuated inflammasome assembly and downstream inflammation, a remodeling shift toward formation, and improved bone microarchitecture and mechanical strength. These findings support the TMAO-linked α-Klotho-TXNIP/NLRP3 axis as a potentially important therapeutic target connecting gut metabolism to skeletal aging.

### Limitations of the study

This study has several limitations. First, the clinical arm was small, single-center, and cross-sectional, limiting statistical power, generalizability, and causal inference regarding associations among exercise, TMAO, inflammation, and bone metabolism. Second, dietary intake was not systematically assessed; in particular, red meat and other choline- or L-carnitine-rich foods, which strongly affect TMAO levels, were not quantified, leaving possible residual dietary confounding. Third, physical activity was classified by self-reported habitual activity rather than a standardized exercise prescription. Exercise type and intensity were not fixed, the lower activity group was not strictly sedentary, and dose-response effects were not assessed; thus, the human data should be interpreted as exploratory associations rather than evidence of a defined exercise effect or optimal exercise volume. Fourth, mechanistic inference regarding the α-Klotho-TXNIP/NLRP3 axis and NLRP3-ASC-caspase-1 complex relied on pharmacological and expression-based manipulations rather than cell-type-specific conditional knockout models. Although IL-1β and IL-18 secretion assays were added to support downstream inflammasome output, more distal pathways such as HMGB1/TLR4 signaling were not examined. The bone turnover panel was also limited to PINP and CTX and did not include bone-specific alkaline phosphatase, osteocalcin, urinary NTX-I, or TRACP5b; bone phenotyping was endpoint based without dynamic histomorphometry. In addition, the D-galactose model only partially reproduces natural skeletal aging, and the use of male rats may limit extrapolation to postmenopausal osteoporosis because sex differences and estrogen status may affect oxidative stress, inflammatory signaling, osteoblast-osteoclast coupling, and α-Klotho-TXNIP/NLRP3 regulation. Fifth, although our findings support a functional association between TMAO and the α-Klotho-TXNIP/NLRP3 axis, they do not establish TMAO as a necessary mediator of exercise-induced bone protection. Further *in vivo* studies using microbiota manipulation, inhibition of TMA/TMAO generation, or TMAO depletion in exercised animals are needed. Plasma and femoral marrow TMAO were also interpreted as complementary, not directly equivalent, exposure indices. Future studies should validate these findings in naturally aged, female, and ovariectomized models and integrate standardized exercise protocols, broader bone phenotyping, advanced genetic models, multi-omics analyses, and larger longitudinal clinical cohorts with detailed dietary assessment.

## Resource availability

### Lead contact

Further information and requests for resources and reagents should be directed to and will be fulfilled by the lead contact, Weijun Gong (gwj197104@ccmu.edu.cn).

### Materials availability

Plasmids and other study-generated materials are available from the lead contact upon reasonable request, subject to institutional regulations and material transfer requirements.

### Data and code availability

• The numerical source data underlying the main and supplemental quantitative figures have been deposited in Zenodo and are publicly available at https://doi.org/10.5281/zenodo.21304790. Unedited and uncropped western blot and co-immunoprecipitation images and representative microscopy and imaging source images are provided as Data S1 and S2, respectively, in the supplemental information.
• This article does not report original code.
• Any additional information required to reanalyze the data reported in this paper is available from the lead contact upon reasonable request.

## Acknowledgments

This work was supported by the 10.13039/501100012166National Key Research and Development Program of China (grant nos. 2023YFC3603800 and 2023YFC3603804).

## Author contributions

Conceptualization, L.Z., X.H., and W.G.; methodology, L.Z. and X.H.; investigation, J.Z., X.S., Q.W., and H.Z.; data curation and formal analysis, L.Z., X.H., and J.Z.; supervision and project administration, W.G.; funding acquisition, W.G.; writing – original draft, L.Z. and X.H.; writing – review and editing, all authors. L.Z. and X.H. contributed equally. All authors approved the final manuscript.

## Declaration of interests

The authors declare no competing interests.

## STAR★Methods

### Key resources table

**Table:** 

REAGENT or RESOURCE	SOURCE	IDENTIFIER
**Antibodies**		

Rabbit anti-TXNIP	Cell Signaling Technology	Cat# 14715; RRID:AB_2714178
Rabbit anti-NLRP3	Cell Signaling Technology; Proteintech for immunofluorescence	CST Cat# 15101; RRID:AB_2722591; Proteintech Cat# 19771-1-AP; RRID:AB_10646484
Rabbit anti-caspase-1	Cell Signaling Technology	Cat# 24232; RRID:AB_2890194
Rabbit anti-α-Klotho	Abcam	Cat# ab181373;RRID:AB_3694098
Rabbit anti-PINP / Procollagen I N-Peptide	Thermo Fisher Scientific	Cat# PA5-35379; RRID:AB_2552689
Rabbit anti-ASC/PYCARD	AdipoGen	Cat# AG-25B-0006-C100; clone AL177; RRID:AB_2490440
Mouse anti-GAPDH	Proteintech	Cat# 60004-1-Ig; RRID:AB_2107436
Anti-NLRP3 antibody for immunoprecipitation	Santa Cruz Biotechnology	Cat# sc-134306; RRID:AB_2152550
Anti-ASC antibody for immunoprecipitation	Santa Cruz Biotechnology	Cat# sc-514414; RRID:AB_2737351
Anti-caspase-1 antibody for immunoprecipitation	Santa Cruz Biotechnology	Cat# sc-392736; RRID:AB_2890615
HRP-conjugated goat anti-rabbit IgG H&L	Abcam	Cat# ab97051; RRID:AB_10679369
HRP-conjugated goat anti-mouse IgG H&L	Abcam	Cat# ab6789; RRID:AB_955439
Alexa Fluor 488 goat anti-rabbit IgG	Invitrogen / Thermo Fisher Scientific	Cat# A-11008; RRID:AB_143165
Alexa Fluor 594 goat anti-mouse IgG	Invitrogen / Thermo Fisher Scientific	Cat# A-11005; RRID:AB_2534073

**Biological samples**		

Human plasma samples	This paper	Samples from adults aged >=65 years
Human fecal samples	This paper	Samples from adults aged >=65 years
Rat plasma samples	This paper	Samples from experimental Sprague-Dawley rats
Rat femoral marrow washouts	This paper	Samples from experimental Sprague-Dawley rats
Rat femoral bone tissue	This paper	Samples from experimental Sprague-Dawley rats

**Experimental models: Organisms/strains**		

Male Sprague-Dawley rats, 6 months old	Beijing Vital River Laboratory Animal Technology	Sprague-Dawley rat; male; 6 months old; vendor strain identifier: SD

**Experimental models: Cell lines**		

Rat osteoblast-like ROS17/2.8 cells	Shanghai Institute of Cell Biology, Chinese Academy of Sciences	Cellosaurus RRID:CVCL_0508

**Chemicals, peptides, and recombinant proteins**		

D-galactose	Sigma-Aldrich	Cat# G0750
Trimethylamine N-oxide (TMAO)	MedChemExpress	Cat# HY-108915; Trimethylamine N-oxide dihydrate
3,3-Dimethyl-1-butanol (DMB)	MedChemExpress	Cat# HY-W012977
MCC950	MedChemExpress	Cat# HY-12815A; MCC950 sodium
Lipopolysaccharide (LPS)	Sigma-Aldrich	Cat# L2630; *E. coli* O111:B4
d9-TMAO internal standard	Cambridge Isotope Laboratories	Cat# DLM-4779; Trimethylamine N-oxide-d9
TRIzol reagent	Invitrogen	Cat# 15596026
DNase I	Thermo Fisher Scientific	Cat# EN0521
HiScript III RT SuperMix	Vazyme	Cat# R323-01
ChamQ Universal SYBR qPCR Master Mix	Vazyme	Cat# Q711-02
Lipofectamine 3000	Invitrogen	Cat# L3000015
Protein A/G agarose beads	Thermo Fisher Scientific	Cat# 20421
Ham's F-12 medium	Gibco	Cat# 11765054
Fetal bovine serum	Gibco	Cat# 10099141C
Penicillin-streptomycin	Gibco	Cat# 15140122

**Critical commercial assays**		

Rat PINP ELISA kit	Elabscience	E-EL-R1414
Rat CTX ELISA kit	Elabscience	E-EL-R1456
Human PINP ELISA kit	Elabscience	E-EL-H0185
Human CTX ELISA kit	Elabscience	E-EL-H0835
Human IL-18 ELISA kit	Elabscience	E-EL-H0253
Human IL-1beta ELISA kit	Elabscience	E-EL-H0149
Rat IL-1beta ELISA kit	Elabscience	E-EL-R0012
Rat IL-18 ELISA kit	Elabscience	E-EL-R0567
Rat RANKL ELISA kit	Jianglai	JL12091-48T
Rat OPG ELISA kit	Elabscience	E-EL-R3005
SA-beta-gal staining kit	Beyotime	C0602
CCK-8 assay kit	Beyotime	Cat# C0037
RayBiotech Rat Cytokine Antibody Array	RayBiotech	Cat# AAR-CYT-1

**Oligonucleotides**		

α-Klotho siRNA	GenePharma	5'-GCCUACUCAUUGUCUUAA-3'
TXNIP siRNA	GenePharma	5'-GCCACUUAUUGAUGUCAA-3'
Non-targeting siRNA control	GenePharma	Negative control siRNA; sequence: 5'-UUCUCCGAACGUGUCACGUTT-3'
qPCR primer: α-Klotho forward	This paper	5'-AGTATGGAGACCTCCCGATG-3'
qPCR primer: α-Klotho reverse	This paper	5'-TCGATAAAAGCCGGACTTGG-3'
qPCR primer: TXNIP forward	This paper	5'-TACAGGTGAGAACGAGATGGTGA-3'
qPCR primer: TXNIP reverse	This paper	5'-TTGAGTTGGCTGGCTGGGAC-3'
qPCR primer: NLRP3 forward	This paper	5'-AAAGCAGCAGATGGAGACTGGAAAG-3'
qPCR primer: NLRP3 reverse	This paper	5'-TGGCAGGTAGGCAGAGAAGAGG-3'
qPCR primer: ASC forward	This paper	5'-ACAGTACCAGGCAGTTCGTG-3'
qPCR primer: ASC reverse	This paper	5'-GGTCTGTCACCAAGTAGGGC-3'
qPCR primer: Caspase-1 forward	This paper	5'-GAAACGCCATGGCTGACAAG-3'
qPCR primer: Caspase-1 reverse	This paper	5'-CATGATCGCACAGGTCTCGT-3'
qPCR primer: GAPDH forward	This paper	5'-AACGACCCCTTCATTGACCTC-3'
qPCR primer: GAPDH reverse	This paper	5'-CGCCAGTAGACTCCACGACATA-3'

**Recombinant DNA**		

pcDNA3.1-α-Klotho plasmid	This paper	Full-length rat α-Klotho cDNA cloned into pcDNA3.1
pcDNA3.1-TXNIP plasmid	This paper	Full-length rat TXNIP cDNA cloned into pcDNA3.1
Empty pcDNA3.1 vector	Invitrogen	pcDNA3.1(+) mammalian expression vector; Cat# V79020

**Software and algorithms**		

SPSS	IBM	Version 27.0
GraphPad Prism	GraphPad Software	Version 9.0
ImageJ	National Institutes of Health	Version 1.53t
QuantStudio software	Applied Biosystems	QuantStudio Design and Analysis Software v1.5.2
Instrument software for mechanical testing	Instron	Bluehill Universal Software v4.0

**Deposited data**		

Numerical source data supporting the main and supplemental quantitative figures	Zenodo	https://doi.org/10.5281/zenodo.21304790

**Other**		

DXA system	GE Healthcare	Lunar Prodigy DXA system
UHPLC-MS/MS system	SCIEX / Shimadzu	SCIEX Triple Quad 5500+ coupled to Shimadzu Nexera X2 UHPLC
BEH Amide column	Waters	ACQUITY UPLC BEH Amide column, 2.1 × 100 mm, 1.7 μm; Cat# 186004801
Motorized treadmill	TSE Systems, Germany	TSE Systems rat treadmill; model 303401
Universal testing machine	Instron	5943
Load cell	Instron	500 N load cell
Micro-CT system	Bruker	SkyScan 1176 micro-CT system
QuantStudio 5 real-time PCR system	Applied Biosystems	QuantStudio 5
Whole-slide scanning system	3DHISTECH	Pannoramic MIDI II whole-slide scanner
Fluorescence or confocal microscope	Leica Microsystems	Leica TCS SP8 confocal microscope

### Experimental model and study participant details

#### Human participants

The clinical component was approved by the Ethics Committee of Beijing Rehabilitation Hospital, Capital Medical University (protocol No. 2024bkky-066). All procedures involving human participants were conducted in accordance with the Declaration of Helsinki and relevant institutional and national regulatory standards. Written informed consent was obtained from all participants before enrollment. The clinical component was designed as an exploratory cross-sectional observational comparison rather than a standardized exercise intervention trial.

Volunteers aged ≥65 years were recruited to investigate the relationship between bone mass and habitual physical activity. Age and sex were recorded for all participants and are reported in Table 1. The study included 20 Chinese older adults, including 10 participants in the higher-activity group and 10 participants in the lower-activity group. Sex distribution was balanced between groups, with 3 males and 7 females in each group. Ethnicity was recorded as Chinese Han for all included participants. Given the exploratory sample size and balanced sex distribution between groups, sex- or gender-stratified analyses were not performed.

#### Animals

Animal procedures were approved by the Ethics Committee of Beijing Rehabilitation Hospital, Capital Medical University (protocol No. 2024bkky-024). All animal experiments were conducted in accordance with the Guide for the Care and Use of Laboratory Animals and relevant institutional and national regulatory standards. Male Sprague-Dawley rats, 6 months old, were obtained from Beijing Vital River Laboratory Animal Technology. Male rats were used in the animal experiments. Animals were housed under specific pathogen-free conditions with a 12:12 h light/dark cycle and *ad libitum* access to food and water. Body weight was recorded every 3 days.

The aging model was established by daily intraperitoneal injection of D-galactose at 200 mg/kg for 12 weeks. Control animals received an equivalent volume of 0.9% saline. Fifty rats were randomly assigned to five groups: Control, Aging model, Exercise, TMAO, and TMAO + Exercise. The Aging model group received D-galactose; the Exercise group received D-galactose plus treadmill training; the TMAO group received D-galactose plus TMAO; the TMAO + Exercise group received D-galactose plus TMAO and treadmill training; and the Control group received saline.

#### Cell lines

Rat osteoblast-like ROS17/2.8 cells were obtained from the Shanghai Institute of Cell Biology, Chinese Academy of Sciences. The recorded sex for this established cell line was not available from the supplier or Cellosaurus. Cells were cultured in Ham’s F-12 medium supplemented with 10% fetal bovine serum and 1% penicillin-streptomycin at 37°C in a humidified incubator with 5% CO2. Cells were passaged using 0.25% trypsin-EDTA at 70%–80% confluence. Cells were used at low passage and were routinely screened for mycoplasma contamination before experiments.

### Method details

#### Clinical grouping and bone phenotyping

The clinical component assessed associations among habitual activity, TMAO exposure, inflammatory cytokines, bone turnover markers, and bone mineral density. Primary outcomes were bone mineral density at the lumbar spine (L1-L4), femoral neck, and total hip. Bone status was classified according to World Health Organization criteria: normal bone mass was defined as T-score ≥ -1.0, osteopenia as -2.5 < T-score < -1.0, and osteoporosis as T-score ≤ -2.5. Secondary outcomes included serum levels of PINP, CTX, IL-1β, and IL-18, as well as TMAO levels in both serum and feces.

#### DXA measurements

Bone mineral density was assessed using dual-energy X-ray absorptiometry at the anteroposterior lumbar spine (L1-L4), femoral neck, and total hip. Scans were acquired by a single trained technologist following standard protocols. Images were analyzed using the manufacturer's software with manual verification. Vertebrae showing marked degeneration, compression, or artifacts were excluded from lumbar spine analysis. T-scores were calculated according to the reference database. Participants, including postmenopausal women and men aged ≥50 years, were classified as normal, osteopenic, or osteoporotic according to the T-score thresholds described above.

#### Rat aging model, TMAO administration, and treadmill exercise

The D-galactose-induced aging model was generated by daily intraperitoneal injection of D-galactose at 200 mg/kg for 12 weeks. TMAO was administered once daily by intragastric gavage at 200 mg/kg/day for 12 weeks. The intragastric route was selected because TMAO is a diet- and gut-related metabolite, and controlled gastrointestinal administration provides reproducible systemic exposure compared with *ad libitum* administration in drinking water. The D-galactose dose and 12-week duration were selected based on established accelerated-aging protocols, whereas the TMAO dose and intervention duration were chosen according to previous *in vivo* TMAO studies and to match the full aging and exercise intervention period.

Exercise training was performed using a motorized treadmill. Training began at 15 m/min for 5 min per session, twice daily, 5 days/week. Intensity was increased weekly by 5 m/min and 5 min until week 3. From week 3 to week 12, the regimen was maintained at 25 m/min for 10 min per session, twice daily, 5 days/week.

#### Sample collection

For human participants, fasting morning K2-EDTA blood samples were collected and processed to plasma within 30 min at 4°C. Freshly voided fecal samples were collected and weighed. For rats, plasma was collected under anesthesia. Immediately after euthanasia, femoral marrow washouts were collected by flushing both femora with cold PBS for UHPLC-MS/MS quantification. Femoral bones were then allocated for biomechanical testing, micro-CT, histology, and molecular assays according to the prespecified experimental design.

#### UHPLC-MS/MS quantification of TMAO

TMAO was measured by UHPLC-MS/MS in human plasma and feces and in rat plasma and femoral marrow. Aliquots of 50 μL plasma or 50–100 mg feces or femoral marrow sample were extracted with prechilled 80% methanol containing d9-TMAO as an internal standard. Samples were vortexed, sonicated on ice, and centrifuged at 15,000 × g for 15 min at 4°C. Supernatants were filtered through 0.22 micrometer membranes before injection.

Chromatographic separation was performed using hydrophilic interaction liquid chromatography on a BEH Amide column, 2.1 × 100 mm, 1.7 μm, at 40°C and a flow rate of 0.30 mL/min with a gradient of acetonitrile and aqueous 10 mM ammonium formate. Detection was performed in positive-ion electrospray ionization mode using triple-quadrupole multiple-reaction monitoring. Quantification was performed using external calibration with internal-standard correction. Plasma TMAO levels are reported as ng/mL, and fecal or femoral marrow TMAO levels are reported as ng/g wet weight.

#### Quality control for UHPLC-MS/MS

Method performance was monitored in each analytical batch using solvent blanks, matrix blanks, calibration standards, internal-standard response checks, and low-, medium-, and high-concentration quality control samples. Calibration curves were generated using weighted linear regression with internal-standard correction and were accepted only when the correlation coefficient was ≥0.99. The lower limit of quantification, intra-assay precision, inter-assay precision, extraction recovery, and carryover were evaluated before sample analysis. Accuracy and precision were required to be within ±15% for non-lower-limit-of-quantification concentrations and within ±20% at the lower limit of quantification. Samples exceeding the calibration range were diluted and reanalyzed. Blank injections were inserted between high-concentration standards or samples to monitor carryover.

#### ELISA

Bone turnover markers, inflammatory cytokines, and osteoblast-derived paracrine factors were measured by ELISA in human samples, rat serum, and cell culture supernatants. Rat serum PINP and CTX were measured using Elabscience kits E-EL-R1414 and E-EL-R1456. In the human cohort, serum PINP and CTX were measured using Elabscience kits E-EL-H0185 and E-EL-H0835. Human IL-18 and IL-1β were measured using Elabscience kits E-EL-H0253 and E-EL-H0149 in paired serum and fecal supernatants.

For *in vitro* experiments, ROS17/2.8 culture supernatants were collected after treatment, centrifuged to remove debris, aliquoted, and stored at -80°C until analysis. Rat IL-1β and IL-18 in culture supernatants were measured using Elabscience kits E-EL-R0012 and E-EL-R0567. RANKL and OPG in culture supernatants were measured using a rat RANKL ELISA kit and a rat OPG ELISA kit, respectively. The RANKL/OPG ratio was calculated for each matched biological replicate.

All ELISAs were performed according to the manufacturers' instructions with blanks, standards, and duplicate wells. Absorbance was read at 450 nm, and concentrations were calculated using four-parameter logistic standard curves. Duplicate coefficients of variation were required to be <15%. Results are reported as serum PINP and CTX in ng/mL; IL-1β, IL-18, RANKL, and OPG in pg/mL; fecal markers normalized to wet weight; and RANKL/OPG as a unitless ratio.

#### Three-point bending of femora

Mechanical properties were assessed by three-point bending. After anesthesia and euthanasia, femora were excised and cleared of soft tissue. For each rat, one femur was assigned to mechanical testing, with left or right side determined by a prespecified randomization scheme, and the contralateral femur was reserved for imaging and histology. The femur assigned to mechanical testing was wrapped in PBS-moistened gauze and stored at 4°C, then equilibrated to room temperature immediately before testing.

Cross-sectional geometry at the mid-diaphysis was obtained by micro-CT on the same unfixed femur before loading using a short scan centered at the mid-diaphysis. These data were used by the instrument software to compute elastic modulus and bending stress based on beam theory. Three-point bending tests were performed on an Instron 5943 universal testing machine equipped with a 500 N load cell and a three-point fixture with a 20 mm span and 2-3 mm radii for the loading nose and supports. The posterior surface rested on the supports with the anterior surface facing up, and the loading point was centered at the mid-diaphysis. After a 1 N preload, displacement-controlled loading proceeded at 1 mm/min to failure while load-displacement curves were recorded.

The following metrics were extracted: maximum load, maximum displacement, bending stiffness as the slope of the linear region, elastic modulus, and bending stress. All tests were performed at room temperature with specimens kept moist throughout. Instruments were calibrated daily. Tests with slippage or atypical support-site fractures were excluded and repeated after repositioning.

#### Micro-CT of trabecular bone

Trabecular micro-CT was performed on the contralateral femur. After euthanasia, femora were cleared of soft tissue, fixed in 4% paraformaldehyde for 24 h, and preserved in 70% ethanol. High-resolution scanning was performed using a Bruker micro-CT system. The distal femoral metaphysis was selected for trabecular analysis. The trabecular region of interest began immediately proximal to the distal growth plate, excluding the primary spongiosa, and extended proximally for 1.0 mm. Cortical bone was manually excluded from the region of interest.

Images were reconstructed and segmented using the same global threshold for all samples within the same analysis batch. The threshold was determined based on visual inspection of representative samples and then kept constant across all groups to avoid group-dependent segmentation bias. Three-dimensional morphometric parameters were calculated, including bone volume fraction, trabecular thickness, trabecular number, and trabecular separation. All region-of-interest selection and morphometric analyses were performed by an investigator blinded to group allocation.

#### Hematoxylin and eosin staining

Following micro-CT imaging, femora were decalcified in 10% EDTA, pH 7.4, at 4°C until fully demineralized. Samples were embedded in paraffin and sectioned coronally at 5 micrometers. Sections were stained with hematoxylin and eosin and imaged using a whole-slide scanning system. The region of interest was standardized to the metaphyseal trabecular bone proximal to the distal growth plate, excluding cortical bone.

#### Rat cytokine antibody array

Serum inflammatory cytokines were profiled using the RayBiotech Rat Cytokine Antibody Array according to the manufacturer's instructions. Blood was collected, clotted at room temperature for 30 min, and centrifuged at 3,000 × g for 10 min. Serum was aliquoted and stored at -80°C. For analysis, samples were thawed once and applied to membranes. After blocking, membranes were incubated with serum overnight at 4°C, followed by sequential incubations with biotinylated detection antibodies and HRP-streptavidin. Signals were visualized by enhanced chemiluminescence and quantified using ImageJ. Data were normalized to on-membrane positive controls after local background subtraction.

#### Cellular senescence induction and treatment

To establish the *in vitro* senescence model, ROS17/2.8 cells were exposed to graded concentrations of D-galactose, ranging from 0–100 mM, for 24–72 h. Cell viability was assessed using CCK-8 assays, and senescence was assessed using SA-β-gal staining. Treatment with 100 mM D-galactose for 72 h was selected for subsequent experiments because it produced a reproducible senescence phenotype while maintaining sufficient cell viability for downstream assays.

Subsequent experiments included four principal groups: Control, Aging model, TMAO, and DMB treatment. The Control group was maintained in normal medium. The Aging model group was treated with 100 mM D-galactose. The TMAO group was treated with 100 mM D-galactose plus 200 micromolar TMAO. The DMB treatment group was treated with 100 mM D-galactose plus 10 mM DMB. DMB was used as an inhibitor of microbial trimethylamine formation. TMAO and DMB concentrations were chosen according to previous *in vitro* studies and preliminary compatibility testing in ROS17/2.8 cells, with the aim of inducing metabolite-related stress or intervention effects without excessive nonspecific cytotoxicity. For pathway perturbation experiments, LPS and MCC950 were applied under established NLRP3 activation and inhibition conditions for 24 h. All experiments included at least three biological replicates with random assignment and blinded analysis.

#### SA-β-gal staining

Cellular senescence was assessed using an SA-β-gal staining kit according to the manufacturer's instructions. Cells were fixed for 15 min and incubated with staining solution at 37°C without CO2 for 12 h. Cells exhibiting blue staining were scored as positive. Bright-field images were acquired from at least five random fields per well. Two independent observers, blinded to treatment allocation, quantified ≥500 cells per sample. The senescence rate was calculated as the percentage of SA-β-gal-positive cells relative to the total cell count.

#### Western blot

Total protein was extracted using RIPA buffer containing protease and phosphatase inhibitors. Lysates were clarified by centrifugation at 12,000 × g for 15 min at 4°C and quantified using a BCA assay. Protein samples were resolved by 8%–12% SDS-PAGE and transferred to PVDF membranes. Membranes were blocked with 5% non-fat milk and incubated overnight at 4°C with the following primary antibodies: rabbit anti-TXNIP, anti-NLRP3, and anti-caspase-1 at 1:1000; rabbit anti-α-Klotho and anti-PINP at 1:1000; mouse anti-ASC/PYCARD at 1:1000; and mouse anti-GAPDH at 1:10,000. After incubation with HRP-conjugated secondary antibodies at 1:10,000, bands were visualized by enhanced chemiluminescence. Protein abundance was quantified using ImageJ and normalized to GAPDH. Data represent at least three independent experiments. Molecular weight standards were included in western blot and co immunoprecipitation experiments. Unedited and uncropped blot images are provided in the supplemental information.

#### qPCR

Total RNA was extracted from cultured cells or pulverized marrow-depleted femoral bone tissue using TRIzol and treated with DNase I. cDNA was synthesized from 1 microliter of total RNA using HiScript III RT SuperMix. qPCR was performed on a QuantStudio 5 system using ChamQ Universal SYBR qPCR Master Mix. Reactions of 10 μL contained 0.2 micromolar primers and 10 ng cDNA. Cycling conditions were 95°C for 30 s, followed by 40 cycles of 95°C for 10 s and 60°C for 30 s. Melting curve analysis was performed to confirm amplification specificity. Samples were analyzed in triplicate, and relative expression was calculated using the 2-delta-delta-Ct method normalized to GAPDH. Primer sequences were as follows: α-Klotho forward, 5'-AGTATGGAGACCTCCCGATG-3'; α-Klotho reverse, 5'-TCGATAAAAGCCGGACTTGG-3'; TXNIP forward, 5'-TACAGGTGAGAACGAGATGGTGA-3'; TXNIP reverse, 5'-TTGAGTTGGCTGGCTGGGAC-3'; NLRP3 forward, 5'-AAAGCAGCAGATGGAGACTGGAAAG-3'; NLRP3 reverse, 5'-TGGCAGGTAGGCAGAGAAGAGG-3'; ASC forward, 5'-ACAGTACCAGGCAGTTCGTG-3'; ASC reverse, 5'-GGTCTGTCACCAAGTAGGGC-3'; Caspase-1 forward, 5'-GAAACGCCATGGCTGACAAG-3'; Caspase-1 reverse, 5'-CATGATCGCACAGGTCTCGT-3'; GAPDH forward, 5'-AACGACCCCTTCATTGACCTC-3'; and GAPDH reverse, 5'-CGCCAGTAGACTCCACGACATA-3'.

#### Co-immunoprecipitation

Protein interactions related to NLRP3 inflammasome assembly were examined by co-immunoprecipitation. Cells were lysed in immunoprecipitation buffer containing 20 mM Tris-HCl, pH 7.4, 150 mM NaCl, 1% NP-40, and protease inhibitors. Lysates were clarified at 14,000 × g for 15 min at 4°C. For each immunoprecipitation reaction, 500 micrograms of lysate was incubated overnight at 4°C with anti-NLRP3, anti-ASC, or anti-caspase-1 antibody at 1:500 under rotation. Protein A/G agarose beads, 40 μL, were added and incubated for 2 h. Complexes were washed, eluted in 2x SDS buffer at 95°C for 10 min, and resolved by SDS-PAGE. Immunoblotting was performed using antibodies against NLRP3, ASC, and caspase-1. Co-immunoprecipitation signals were quantified using ImageJ and normalized to the corresponding immunoprecipitated bait protein or input control, as indicated.

#### Immunofluorescence colocalization analysis

Double-immunofluorescence staining was performed to evaluate spatial colocalization of NLRP3 with ASC or caspase-1 in ROS17/2.8 cells. Cells were seeded on sterile glass coverslips in 24-well plates and assigned to four treatment conditions: Control, D-galactose-induced senescence model, TMAO treatment, and TMAO inhibition treatment. The senescence model was established using 100 mM D-galactose for 72 h. The TMAO group was treated with 100 mM D-galactose plus 200 micromolar TMAO, and the TMAO inhibition group was treated with 100 mM D-galactose plus 10 mM DMB.

After treatment, cells were washed with PBS, fixed with 4% paraformaldehyde for 15 min at room temperature, permeabilized with 0.2% Triton X-100 for 10 min, and blocked with 5% bovine serum albumin for 1 h. Cells were incubated overnight at 4°C with primary antibodies, including rabbit anti-NLRP3 at 1:200, mouse anti-ASC/PYCARD at 1:200, and mouse anti-caspase-1 at 1:200. For NLRP3/ASC colocalization, cells were co-incubated with anti-NLRP3 and anti-ASC/PYCARD antibodies. For NLRP3/caspase-1 colocalization, cells were co-incubated with anti-NLRP3 and anti-caspase-1 antibodies. After washing, cells were incubated with species-appropriate Alexa Fluor-conjugated secondary antibodies at 1:500 for 1 h at room temperature in the dark. Nuclei were counterstained with DAPI, and coverslips were mounted with antifade mounting medium.

Fluorescence images were acquired using a fluorescence or confocal microscope under identical imaging settings across groups. Negative controls without primary antibodies and single-staining controls were included to assess nonspecific secondary antibody signals and potential channel bleed-through. NLRP3/ASC and NLRP3/caspase-1 colocalization was evaluated based on punctate merged fluorescence signals in overlay images, reflecting spatial recruitment of ASC or caspase-1 to NLRP3.

#### Genetic modulation of α-Klotho and TXNIP

To modulate α-Klotho and TXNIP expression, ROS17/2.8 cells were subjected to either siRNA-mediated knockdown or plasmid-mediated overexpression. For knockdown, gene-specific siRNAs targeting α-Klotho or TXNIP were transfected using Lipofectamine 3000. A non-targeting negative-control siRNA was used as the control. Medium was replaced after 6 h, and cells were harvested 48 h after transfection.

For overexpression, full-length rat α-Klotho and TXNIP cDNA sequences were cloned into the pcDNA3.1 vector. Cells were transfected with the respective overexpression plasmids or an empty pcDNA3.1 vector using Lipofectamine 3000. Cells were cultured for 48 h before analysis. Knockdown and overexpression efficiencies for both genes were verified by Western blot and qPCR. siRNA sequences were as follows: α-Klotho, 5'-GCCUACUCAUUGUCUUAA-3'; and TXNIP, 5'-GCCACUUAUUGAUGUCAA-3'.

#### Randomization and blinding

Animals were randomly assigned to experimental groups using a computer-generated randomization sequence after acclimatization. For biomechanical testing, micro-CT scanning, histological processing, ELISA, qPCR, Western blot, co-immunoprecipitation, and image quantification, samples were labeled with coded identifiers. Investigators responsible for outcome measurement, image acquisition, densitometric analysis, and statistical analysis were blinded to group allocation until completion of data analysis. For cellular experiments, wells or culture dishes were randomly assigned to treatment conditions, and image-based quantification, including SA-β-gal positivity and fluorescence or histological measurements, was performed by investigators blinded to treatment identity.

### Quantification and statistical analysis

Clinical data were analyzed using SPSS version 27.0. Continuous variables are presented as mean ± SD, and categorical variables are presented as n (%). Normality was assessed using the Shapiro-Wilk test. Between-group comparisons for continuous variables were performed using an independent-samples t test when data were normally distributed; otherwise, the Mann-Whitney U test was applied. Categorical variables were compared using Fisher's exact test. All tests were two-tailed, with alpha = 0.05, and *p* < 0.05 was considered statistically significant.

Animal and cellular data were analyzed using GraphPad Prism version 9.0 and are presented as mean ± SD. Homogeneity of variance was assessed using Levene's test. Two-group comparisons were performed using Student's t test. Comparisons among three or more groups were performed using one-way ANOVA followed by Sidak's or Tukey's post hoc multiple-comparison correction, as appropriate. *p* < 0.05 was considered statistically significant. The number of biological replicates and exact statistical tests are indicated in the figure legends or corresponding results sections where applicable.

Statistical tests, biological units, sample sizes, and asterisk definitions are provided in the corresponding figure legends. Detailed statistical information for the main and supplemental figures, including statistical tests, biological units, sample sizes, group comparisons, multiple-comparison procedures, test statistics, degrees of freedom, exact *p* values, effect estimates where applicable, and figure-level significance symbols, is provided in Table S1.
