# Supplementary material for: Exercise is associated with attenuated aging-related osteoporosis through TMAO alpha Klotho inflammasome signaling
Source: iScience. 2026 Aug 11;29(8):117170. doi: 10.1016/j.isci.2026.117170 (PMC13486840; doi:10.1016/j.isci.2026.117170)

iScience, Volume *29*

Supplemental information

Exercise is associated with attenuated aging-related osteoporosis through TMAO alpha Klotho inflammasome signaling

Lihua Zhang, Xiaoyu Huang, Jiawei Zhang, Xiangyue Si, Hongchun Zuo, Qun Wang, and Weijun Gong

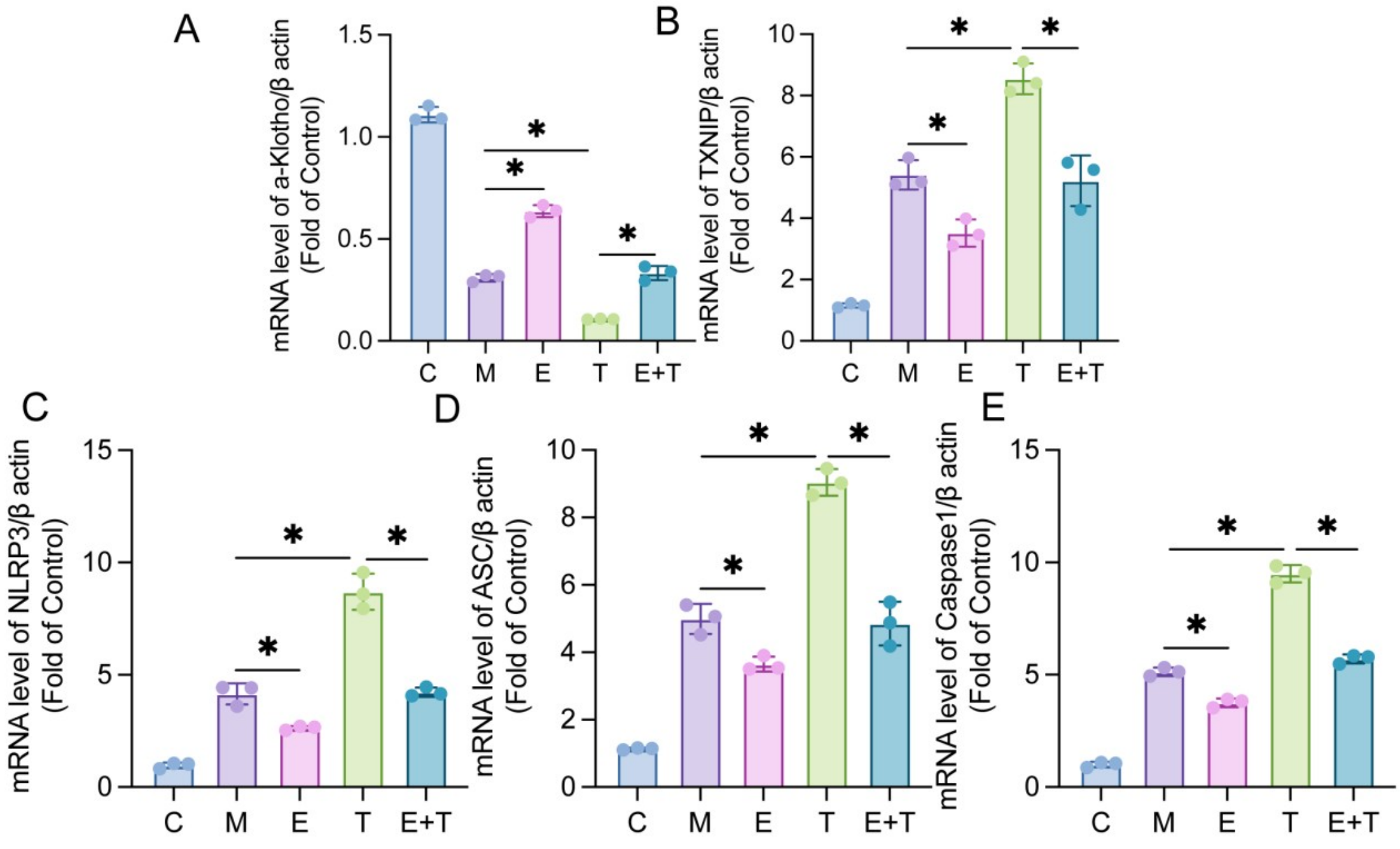

Supplementary Figure 1. RT–qPCR analysis of inflammasome-related transcripts in aged rat bone across five groups.

Relative mRNA levels of α-Klotho (A), TXNIP (B), NLRP3 (C), ASC (D), and Caspase-1 (E) in femoral bone tissue from: control (C), D-galactose aging model (M), exercise (E), TMAO administration (T), and exercise plus TMAO (E+T). Expression was normalized to β-actin and plotted as fold-change versus Control. TMAO elevated TXNIP, NLRP3, ASC, and Caspase-1 transcripts while suppressing α-Klotho; exercise alone shifted these changes toward Control, and concomitant exercise mitigated the TMAO effect. Data are shown as mean ± SD (dots represent individual samples). Statistics: one-way ANOVA with Tukey's multiple-comparison test; *$P < 0.05$ for the indicated pairwise comparisons. Abbreviations: TXNIP, thioredoxin-interacting protein; ASC, apoptosis-associated speck-like protein containing a CARD.

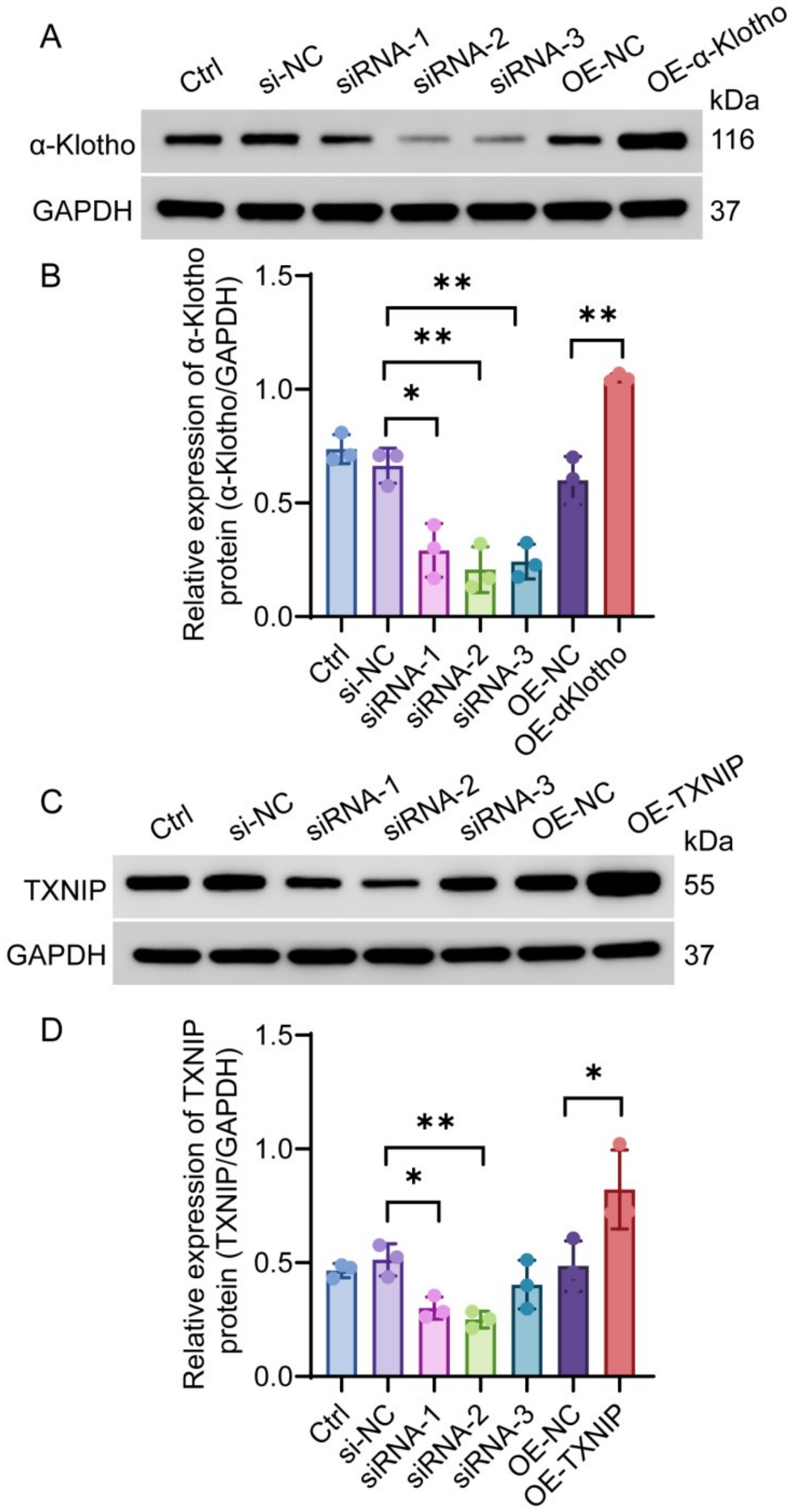

Supplementary Figure 2. Validation of α-Klotho and TXNIP knockdown/overexpression in osteoblast-like cells.

(A–B) Western blots and densitometric quantification of α-Klotho (~116 kDa) after transfection with three independent siRNAs (siRNA-1/2/3), non-targeting control (si-NC), overexpression plasmid (OE-α-Klotho), or empty vector (OE-NC). GAPDH (37 kDa) served as the loading control; values are normalized to GAPDH and expressed relative to the untreated control (Ctrl). α-Klotho siRNAs reduced protein abundance, whereas OE-α-Klotho markedly increased it.

(C–D) Western blots and quantification of TXNIP (~55 kDa) under the same

conditions (siRNA-1/2/3, si-NC, OE-TXNIP, OE-NC). TXNIP siRNAs decreased, and OE-TXNIP increased, TXNIP protein levels. Bars show mean ± SD. Statistics: one-way ANOVA with Tukey's post hoc test; *P < 0.05, **P < 0.01 for the indicated comparisons. The most efficient siRNA for each gene was used in subsequent experiments. Abbreviations: OE, overexpression; NC, negative control.

Table S1. Statistical information

Figure panel	Outcome / analysis	Biological unit	Sample size	Statistical test	Comparison or factor	Multiple-comparison procedure	Test statistic	df	Exact P value	Effect estimate / 95% CI	Figure symbol
Figure 1B	Lumbar spine L1–L4 T-score	participant	Control n = 10; Exercise n = 10	Unpaired two-tailed Student’s t test	Exercise vs Control	None	t = 0.6223	18	0.5416	Mean difference (Exercise-Control) = 0.4800; 95% CI -1.141 to 2.101; R^2 = 0.02106	ns
Figure 1C	Femoral neck T-score	participant	Control n = 10; Exercise n = 10	Unpaired two-tailed Student’s t test	Exercise vs Control	None	t = 2.575	18	0.0191	Mean difference (Exercise-Control) = 1.220; 95% CI 0.2245 to 2.216; R^2 = 0.2692	*
Figure 1D	Total hip T-score	participant	Control n = 10; Exercise n = 10	Unpaired two-tailed Student’s t test	Exercise vs Control	None	t = 2.182	18	0.0426	Mean difference (Exercise-Control) = 1.180; 95% CI 0.04408 to 2.316; R^2 = 0.2092	*
Figure 1E	Fecal TMAO	participant	Control n = 8; Exercise n = 7	Welch’s two-tailed t test	Exercise vs Control	None	t = 2.558	9.459	0.0297	Mean difference (Exercise-Control) = -265.9; 95% CI -499.4 to -32.47; R^2 = 0.4089	*
Figure 1F	Serum TMAO	participant	Control n = 10; Exercise n = 9	Welch’s two-tailed t test	Exercise vs Control	None	t = 2.644	11.94	0.0215	Mean difference (Exercise-Control) = -44.94; 95% CI -81.99 to -7.890; R^2 = 0.3693	*
Figure 1G	Serum PINP	participant	Control n = 10; Exercise n = 10	Unpaired two-tailed Student’s t test	Exercise vs Control	None	t = 3.757	18	0.0014	Mean difference (Exercise-Control) = 43.74; 95% CI 19.28 to 68.20; R^2 = 0.4395	**
Figure 1H	Serum CTX	participant	Control n = 10; Exercise n = 10	Unpaired two-tailed Student’s t test	Exercise vs Control	None	t = 3.738	18	0.0015	Mean difference (Exercise-Control) = -0.4665; 95% CI -0.7287 to -0.2043; R^2 = 0.4370	**
Figure 1I	Serum IL-18	participant	Control n = 10; Exercise n = 10	Unpaired two-tailed Student’s t test	Exercise vs Control	None	t = 6.318	18	<0.0001	Mean difference (Exercise-Control) = -177.6; 95% CI -236.6 to -118.5; R^2 = 0.6892	*
Figure 1J	Fecal IL-18	participant	Control n = 9; Exercise n = 10	Unpaired two-tailed Student’s t test	Exercise vs Control	None	t = 4.957	17	0.0001	Mean difference (Exercise-Control) = -9.759; 95% CI -13.91 to -5.605; R^2 = 0.5911	*
Figure 1K	Serum IL-1β	participant	Control n = 10; Exercise n = 10	Unpaired two-tailed Student’s t test	Exercise vs Control	None	t = 6.081	18	<0.0001	Mean difference (Exercise-Control) = -3.812; 95% CI -5.129 to -2.495; R^2 = 0.6726	*
Figure 1L	Fecal IL-1β	participant	Control n = 9; Exercise n = 10	Unpaired two-tailed Student’s t test	Exercise vs Control	None	t = 5.545	17	<0.0001	Mean difference (Exercise-Control) = -32.68; 95% CI -45.12 to -20.25; R^2 = 0.6440	*
Figure 2B	Maximum load	rat	n = 3 rats per group	One-way ANOVA	Group effect; selected Tukey comparisons	Tukey’s multiple comparisons test	F = 165.4	4, 10	<0.0001	R^2 = 0.9851	*
Figure 2C	Maximum deformation	rat	n = 3 rats per group	One-way ANOVA	Group effect	Tukey’s multiple comparisons test	F = 1.888	4, 10	0.1892	R^2 = 0.4303	ns
Figure 2D	Bending stiffness	rat	n = 3 rats per group	One-way ANOVA	Group effect	Tukey’s multiple comparisons test	F = 0.4324	4, 10	0.7824	R^2 = 0.1475	ns
Figure 2E	Elastic modulus	rat	n = 3 rats per group	One-way ANOVA	Group effect	Tukey’s multiple comparisons test	F = 0.2407	4, 10	0.9089	R^2 = 0.08782	ns
Figure 2F	Bending stress	rat	n = 3 rats per group	One-way ANOVA	Group effect	Tukey’s multiple comparisons test	F = 0.5614	4, 10	0.6962	R^2 = 0.1834	ns
Figure 2H	BV/TV	rat	n = 3 rats per group	One-way ANOVA	Group effect; selected Tukey comparisons	Tukey’s multiple comparisons test	F = 416.7	4, 10	<0.0001	R^2 = 0.9940	*
Figure 2I	Trabecular thickness	rat	n = 3 rats per group	One-way ANOVA	Group effect	Tukey’s multiple comparisons test	F = 0.6412	4, 10	0.6452	R^2 = 0.2041	ns
Figure 2J	Trabecular number	rat	n = 3 rats per group	One-way ANOVA	Group effect; selected Tukey comparisons	Tukey’s multiple comparisons test	F = 284.1	4, 10	<0.0001	R^2 = 0.9913	*
Figure 2K	Trabecular separation	rat	n = 3 rats per group	One-way ANOVA	Group effect; selected Tukey comparisons	Tukey’s multiple comparisons test	F = 500.3	4, 10	<0.0001	R^2 = 0.9950	*
Figure 3A	Femoral marrow TMAO	rat	n = 3 rats per group	One-way ANOVA	Group effect; selected Tukey comparisons	Tukey’s multiple comparisons test	F = 216.5	4, 10	<0.0001	R^2 = 0.9886	*
Figure 3B	Plasma TMAO	rat	n = 3 rats per group	One-way ANOVA	Group effect; selected Tukey comparisons	Tukey’s multiple comparisons test	F = 220.0	4, 10	<0.0001	R^2 = 0.9888	*
Figure 3C	Serum CTX	rat	n = 5 rats per group	One-way ANOVA	Group effect; selected Tukey comparisons	Tukey’s multiple comparisons test	F = 123.6	4, 20	<0.0001	R^2 = 0.9611	*
Figure 3D	Serum PINP	rat	n = 5 rats per group	One-way ANOVA	Group effect; selected Tukey comparisons	Tukey’s multiple comparisons test	F = 169.3	4, 20	<0.0001	R^2 = 0.9713	*
Figure 4A	CCK-8 cell viability, 24 h	independent biological experiment	n = 5 per concentration/time condition	One-way ANOVA	D-galactose concentration at 24 h	Tukey’s multiple comparisons test	F = 30.73	3, 16	<0.0001	R^2 = 0.8521	*
Figure 4A	CCK-8 cell viability, 48 h	independent biological experiment	n = 5 per concentration/time condition	One-way ANOVA	D-galactose concentration at 48 h	Tukey’s multiple comparisons test	F = 188.9	3, 16	<0.0001	R^2 = 0.9725	*
Figure 4A	CCK-8 cell viability, 72 h	independent biological experiment	n = 5 per concentration/time condition	One-way ANOVA	D-galactose concentration at 72 h	Tukey’s multiple comparisons test	F = 114.0	3, 16	<0.0001	R^2 = 0.9553	*
Figure 4B	SA-β-gal-positive cell rate, 24 h	independent biological experiment	n = 3 per concentration/time condition	One-way ANOVA	D-galactose concentration at 24 h	Tukey’s multiple comparisons test	F = 80.27	3, 8	<0.0001	R^2 = 0.9678	*
Figure 4B	SA-β-gal-positive cell rate, 48 h	independent biological experiment	n = 3 per concentration/time condition	One-way ANOVA	D-galactose concentration at 48 h	Tukey’s multiple comparisons test	F = 103.6	3, 8	<0.0001	R^2 = 0.9749	*
Figure 4B	SA-β-gal-positive cell rate, 72 h	independent biological experiment	n = 3 per concentration/time condition	One-way ANOVA	D-galactose concentration at 72 h	Tukey’s multiple comparisons test	F = 150.1	3, 8	<0.0001	R^2 = 0.9825	*
Figure 4C	SA-β-gal staining result after TMAO/DMB	independent biological experiment	n = 3 per group	One-way ANOVA	Group effect; selected Tukey comparisons	Tukey’s multiple comparisons test	F = 308.3	3, 8	<0.0001	R^2 = 0.9914	*, **
Figure 4E	PINP/GAPDH western blot quantification	independent western blot experiment	n = 3 per group	One-way ANOVA	Group effect; selected Tukey comparisons	Tukey’s multiple comparisons test	F = 61.54	3, 8	<0.0001	R^2 = 0.9585	*, ***
Figure 4F	RANKL/OPG ratio	independent biological experiment	n = 6 per group	One-way ANOVA	Group effect; selected Tukey comparisons	Tukey’s multiple comparisons test	F = 790.8	3, 20	<0.0001	R^2 = 0.9916	*, ***
Figure 5A	α-Klotho mRNA expression	independent biological experiment	n = 3 per group	One-way ANOVA	Group effect; selected Tukey comparisons	Tukey’s multiple comparisons test	F = 109.3	3, 8	<0.0001	R^2 = 0.9762	*, **, ***
Figure 5A	TXNIP mRNA expression	independent biological experiment	n = 3 per group	One-way ANOVA	Group effect; selected Tukey comparisons	Tukey’s multiple comparisons test	F = 38.42	3, 8	<0.0001	R^2 = 0.9351	*, **, ***
Figure 5A	NLRP3 mRNA expression	independent biological experiment	n = 3 per group	One-way ANOVA	Group effect; selected Tukey comparisons	Tukey’s multiple comparisons test	F = 164.3	3, 8	<0.0001	R^2 = 0.9840	*, **, ***
Figure 5A	ASC mRNA expression	independent biological experiment	n = 3 per group	One-way ANOVA	Group effect; selected Tukey comparisons	Tukey’s multiple comparisons test	F = 54.07	3, 8	<0.0001	R^2 = 0.9530	*, **, ***
Figure 5A	Caspase-1 mRNA expression	independent biological experiment	n = 3 per group	One-way ANOVA	Group effect; selected Tukey comparisons	Tukey’s multiple comparisons test	F = 220.1	3, 8	<0.0001	R^2 = 0.9880	*, **, ***
Figure 5C	TXNIP/GAPDH western blot quantification	independent western blot experiment	n = 3 per group	One-way ANOVA	Group effect; selected Tukey comparisons	Tukey’s multiple comparisons test	F = 272.9	3, 8	<0.0001	R^2 = 0.9903	*, **, ***
Figure 5D	α-Klotho/GAPDH western blot quantification	independent western blot experiment	n = 3 per group	One-way ANOVA	Group effect; selected Tukey comparisons	Tukey’s multiple comparisons test	F = 21.88	3, 8	0.0003	R^2 = 0.8914	*, **, ***
Figure 5E	NLRP3/GAPDH western blot quantification	independent western blot experiment	n = 3 per group	One-way ANOVA	Group effect; selected Tukey comparisons	Tukey’s multiple comparisons test	F = 34.83	3, 8	<0.0001	R^2 = 0.9289	*, **, ***
Figure 5F	ASC/GAPDH western blot quantification	independent western blot experiment	n = 3 per group	One-way ANOVA	Group effect; selected Tukey comparisons	Tukey’s multiple comparisons test	F = 21.32	3, 8	0.0004	R^2 = 0.8888	*, **, ***
Figure 5G	Caspase-1/GAPDH western blot quantification	independent western blot experiment	n = 3 per group	One-way ANOVA	Group effect; selected Tukey comparisons	Tukey’s multiple comparisons test	F = 37.00	3, 8	<0.0001	R^2 = 0.9328	*, **, ***

Figure panel	Outcome / analysis	Biological unit	Sample size	Statistical test	Comparison or factor	Multiple-comparison procedure	Test statistic	df	Exact P value	Effect estimate / 95% CI	Figure symbol
Figure 5H	IL-1β secretion	independent biological experiment	n = 6 per group	One-way ANOVA	Group effect; selected Tukey comparisons	Tukey's multiple comparisons test	F = 422.2	3, 20	<0.0001	R^2 = 0.9845	*, **, ***
Figure 5I	IL-18 secretion	independent biological experiment	n = 6 per group	One-way ANOVA	Group effect; selected Tukey comparisons	Tukey's multiple comparisons test	F = 789.0	3, 20	<0.0001	R^2 = 0.9916	*, **, ***
Figure 6B	ASC/NLRP3 co-IP quantification	independent co-IP experiment	n = 3 per group	One-way ANOVA	Group effect; selected Tukey comparisons	Tukey's multiple comparisons test	F = 164.1	3, 8	<0.0001	R^2 = 0.9840	*
Figure 6C	NLRP3/ASC co-IP quantification	independent co-IP experiment	n = 3 per group	One-way ANOVA	Group effect; selected Tukey comparisons	Tukey's multiple comparisons test	F = 96.97	3, 8	<0.0001	R^2 = 0.9732	*
Figure 6E	Caspase-1/NLRP3 co-IP quantification	independent co-IP experiment	n = 3 per group	One-way ANOVA	Group effect; selected Tukey comparisons	Tukey's multiple comparisons test	F = 121.0	3, 8	<0.0001	R^2 = 0.9784	*
Figure 6F	NLRP3/Caspase-1 co-IP quantification	independent co-IP experiment	n = 3 per group	One-way ANOVA	Group effect; selected Tukey comparisons	Tukey's multiple comparisons test	F = 188.1	3, 8	<0.0001	R^2 = 0.9860	*
Figure 7B	TXNIP/GAPDH western blot quantification after genetic/pharmacological perturbation	independent western blot experiment	n = 3 per group	One-way ANOVA	Group effect; selected Tukey comparisons	Tukey's multiple comparisons test	F = 35.25	6, 14	<0.0001	R^2 = 0.9379	*
Figure 7C	α-Klotho/GAPDH western blot quantification after genetic/pharmacological perturbation	independent western blot experiment	n = 3 per group	One-way ANOVA	Group effect; selected Tukey comparisons	Tukey's multiple comparisons test	F = 35.89	6, 14	<0.0001	R^2 = 0.9390	*
Figure 7D	SA-β-gal-positive cell rate after genetic/pharmacological perturbation	independent biological experiment	n = 3 per group	One-way ANOVA	Group effect; selected Tukey comparisons	Tukey's multiple comparisons test	F = 138.1	6, 14	<0.0001	R^2 = 0.9834	*
Figure 8B	ASC/NLRP3 co-IP quantification after perturbation	independent co-IP experiment	n = 3 per group	One-way ANOVA	Group effect; selected Tukey comparisons	Tukey's multiple comparisons test	F = 77.29	6, 14	<0.0001	R^2 = 0.9707	*
Figure 8C	NLRP3/ASC co-IP quantification after perturbation	independent co-IP experiment	n = 3 per group	One-way ANOVA	Group effect; selected Tukey comparisons	Tukey's multiple comparisons test	F = 170.5	6, 14	<0.0001	R^2 = 0.9865	*
Figure 8E	Caspase-1/NLRP3 co-IP quantification after perturbation	independent co-IP experiment	n = 3 per group	One-way ANOVA	Group effect; selected Tukey comparisons	Tukey's multiple comparisons test	F = 100.3	6, 14	<0.0001	R^2 = 0.9773	*
Figure 8F	NLRP3/Caspase-1 co-IP quantification after perturbation	independent co-IP experiment	n = 3 per group	One-way ANOVA	Group effect; selected Tukey comparisons	Tukey's multiple comparisons test	F = 85.09	6, 14	<0.0001	R^2 = 0.9739	*
Figure S1A	α-Klotho mRNA expression in rat femoral bone	rat	n = 3 rats per group	One-way ANOVA	Group effect; selected Tukey comparisons	Tukey's multiple comparisons test	F = 580.0	4, 10	<0.0001	R^2 = 0.9957	*
Figure S1B	TXNIP mRNA expression in rat femoral bone	rat	n = 3 rats per group	One-way ANOVA	Group effect; selected Tukey comparisons	Tukey's multiple comparisons test	F = 80.93	4, 10	<0.0001	R^2 = 0.9700	*
Figure S1C	NLRP3 mRNA expression in rat femoral bone	rat	n = 3 rats per group	One-way ANOVA	Group effect; selected Tukey comparisons	Tukey's multiple comparisons test	F = 132.5	4, 10	<0.0001	R^2 = 0.9815	*
Figure S1D	ASC mRNA expression in rat femoral bone	rat	n = 3 rats per group	One-way ANOVA	Group effect; selected Tukey comparisons	Tukey's multiple comparisons test	F = 148.8	4, 10	<0.0001	R^2 = 0.9835	*
Figure S1E	Caspase-1 mRNA expression in rat femoral bone	rat	n = 3 rats per group	One-way ANOVA	Group effect; selected Tukey comparisons	Tukey's multiple comparisons test	F = 517.2	4, 10	<0.0001	R^2 = 0.9952	*
Figure S2B	α-Klotho/GAPDH western blot quantification for knockdown and overexpression validation	independent western blot experiment	n = 3 per group	One-way ANOVA	Group effect; selected Tukey comparisons	Tukey's multiple comparisons test	F = 39.55	6, 14	<0.0001	R^2 = 0.9443	*, **
Figure S2D	TXNIP/GAPDH western blot quantification for knockdown and overexpression validation	independent western blot experiment	n = 3 per group	One-way ANOVA	Group effect; selected Tukey comparisons	Tukey's multiple comparisons test	F = 11.41	6, 14	0.0001	R^2 = 0.8302	*, **

Data S1. Unedited and uncropped western blot and co-immunoprecipitation images.

Figure 4D

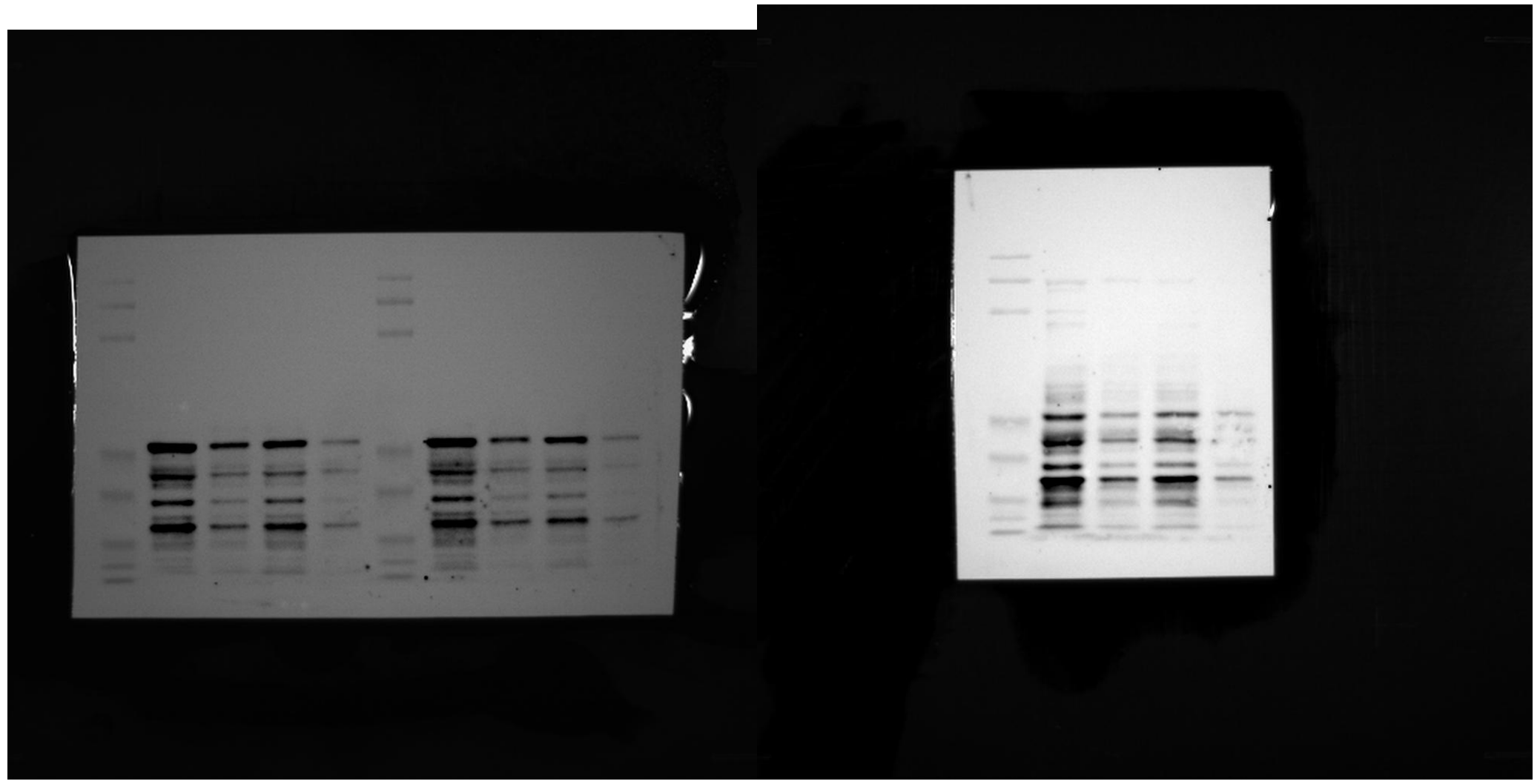

PINP

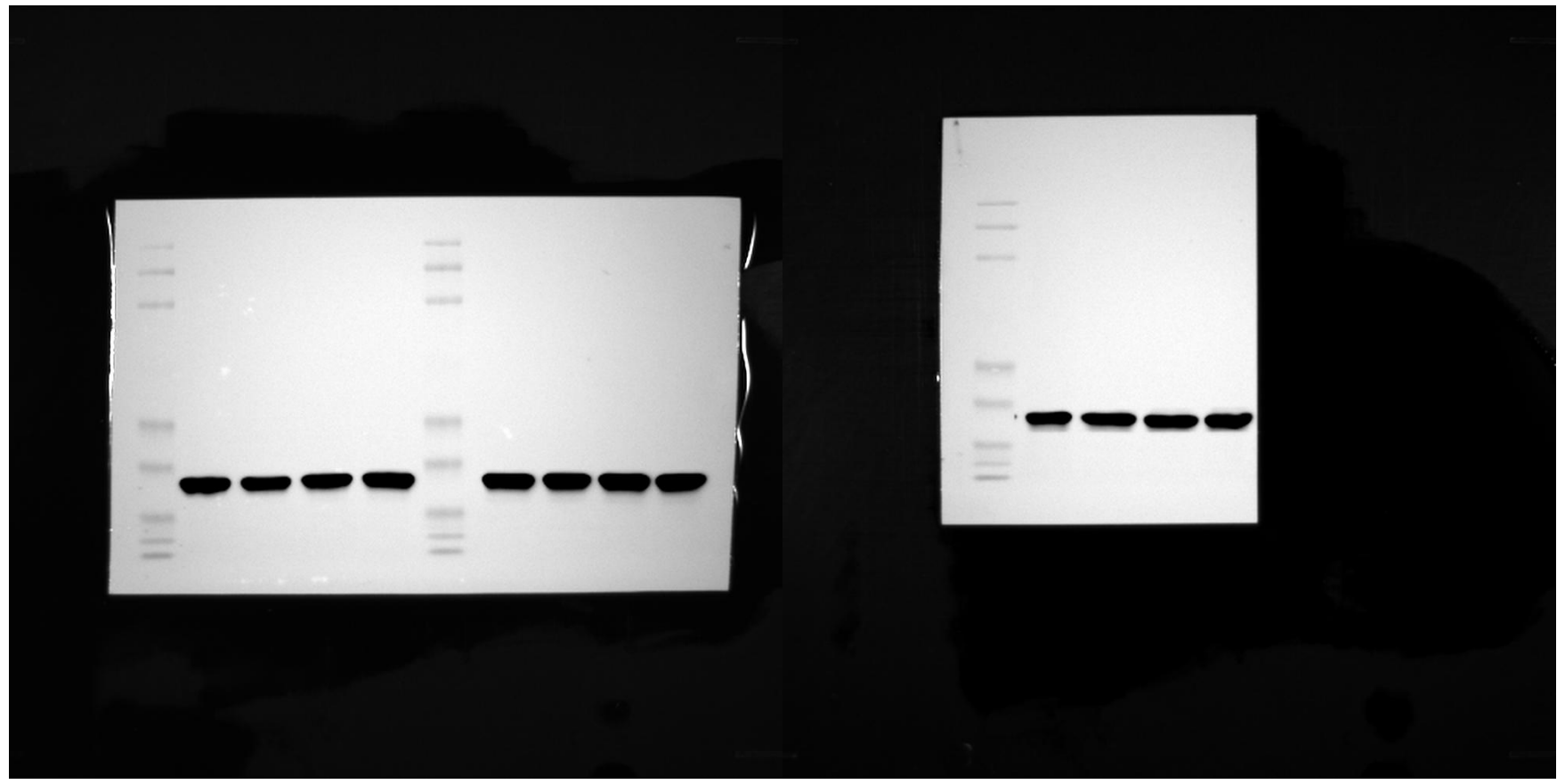

GAPDH

Figure 5B

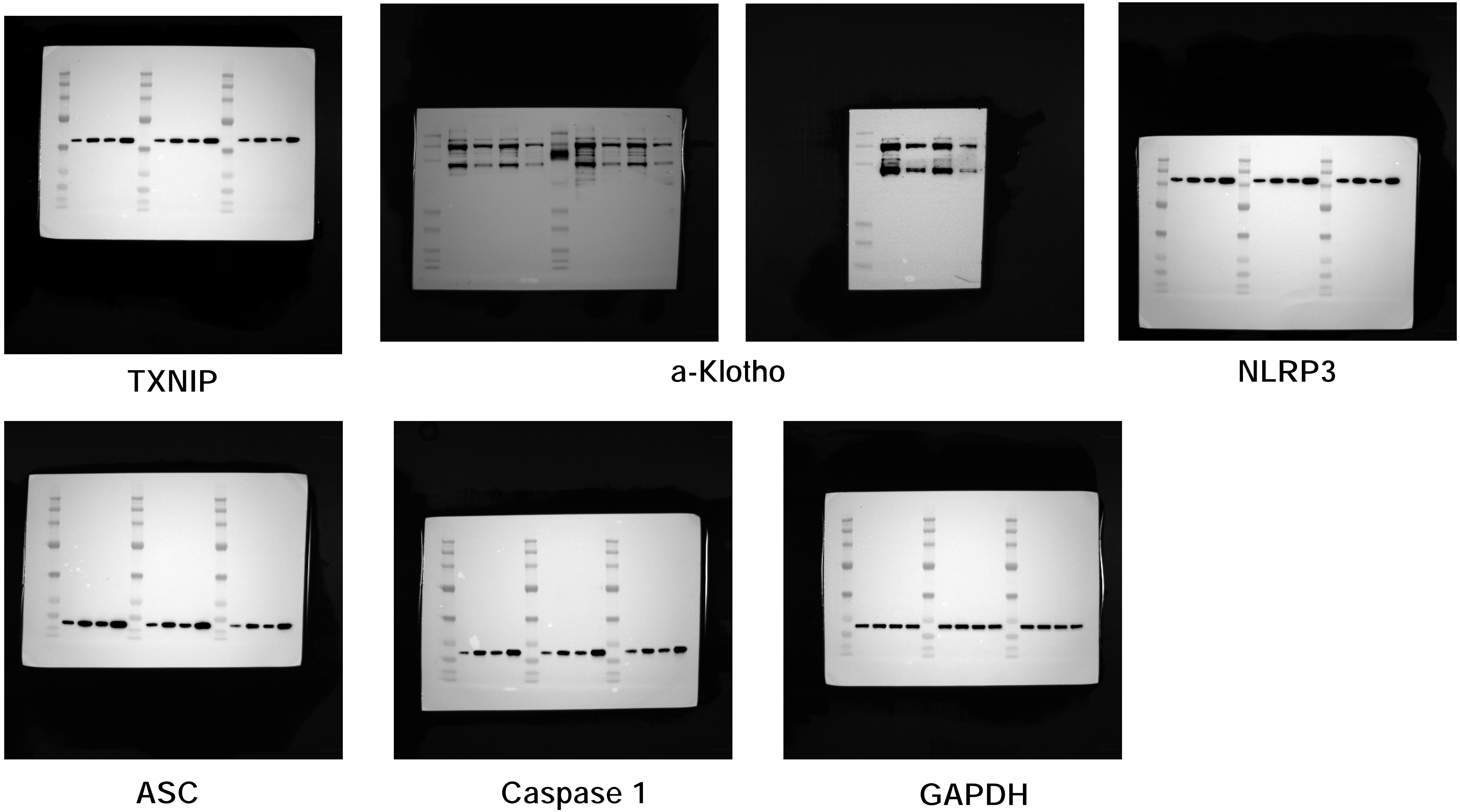

Figure 6A

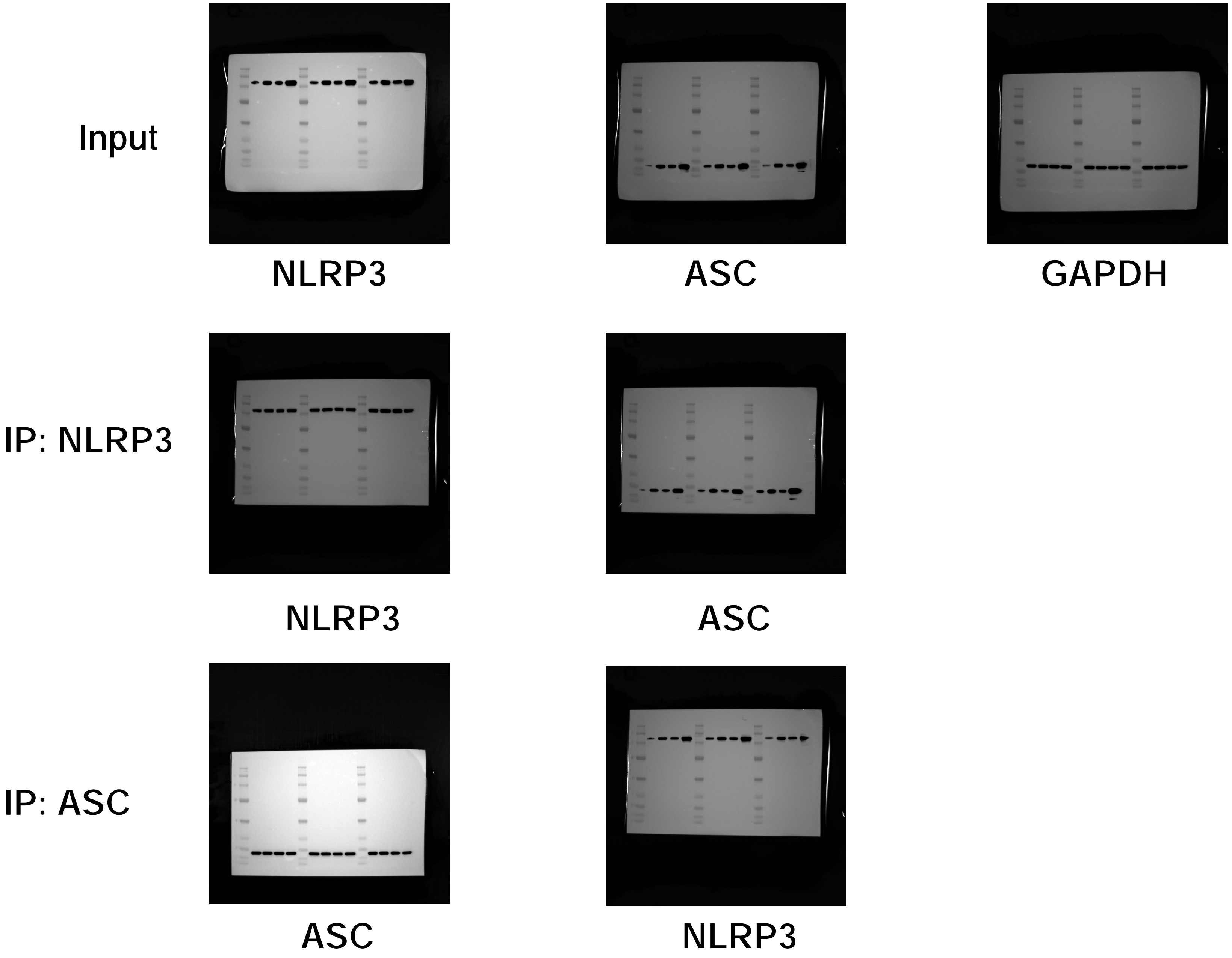

Figure 6D

Input

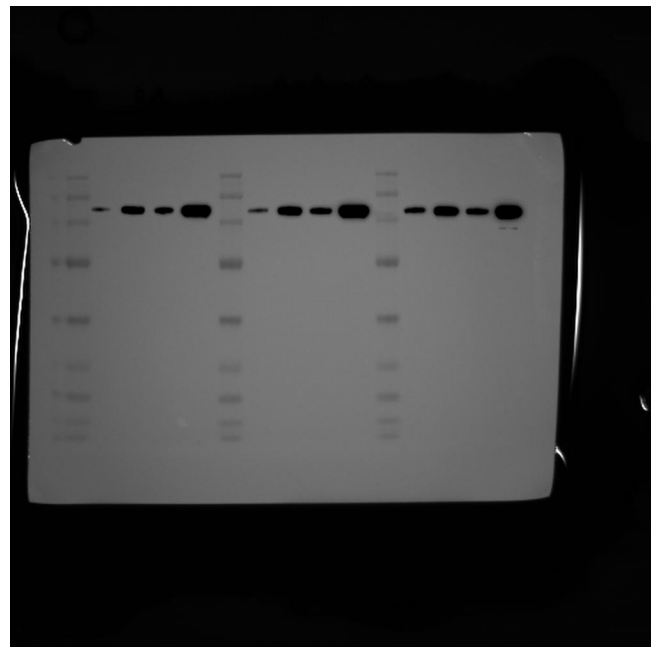

NLRP3

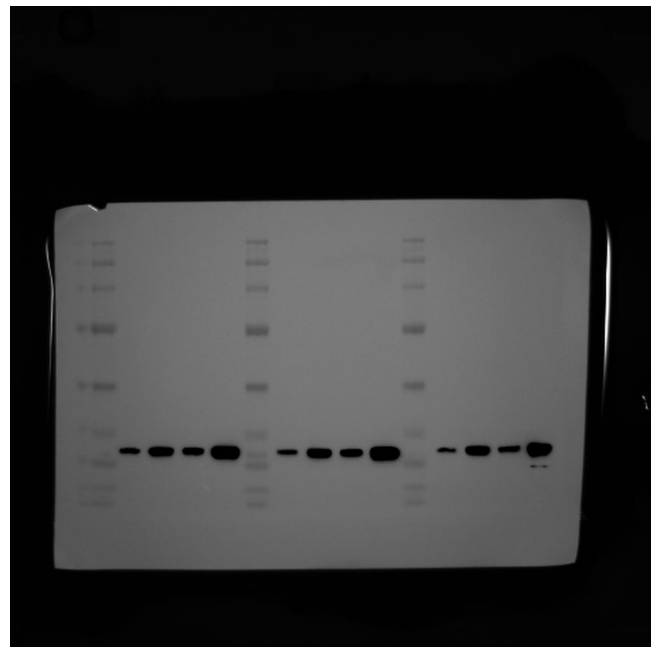

Caspase 1

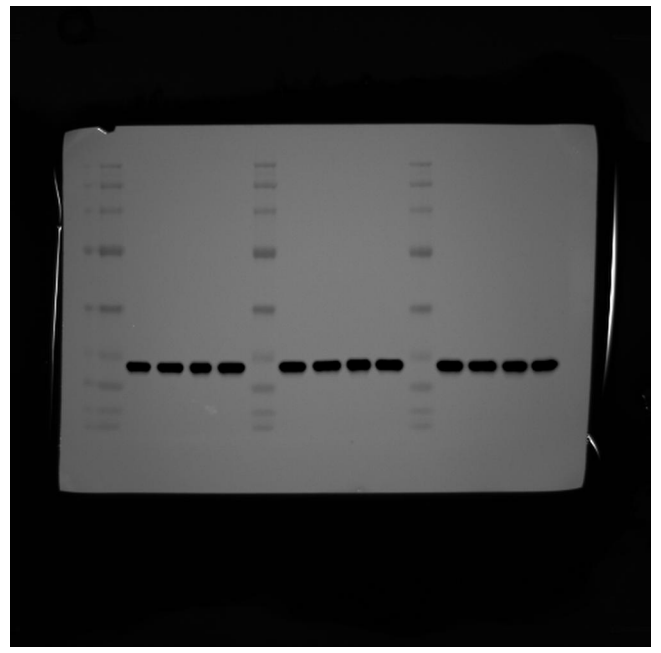

GAPDH

IP: NLRP3

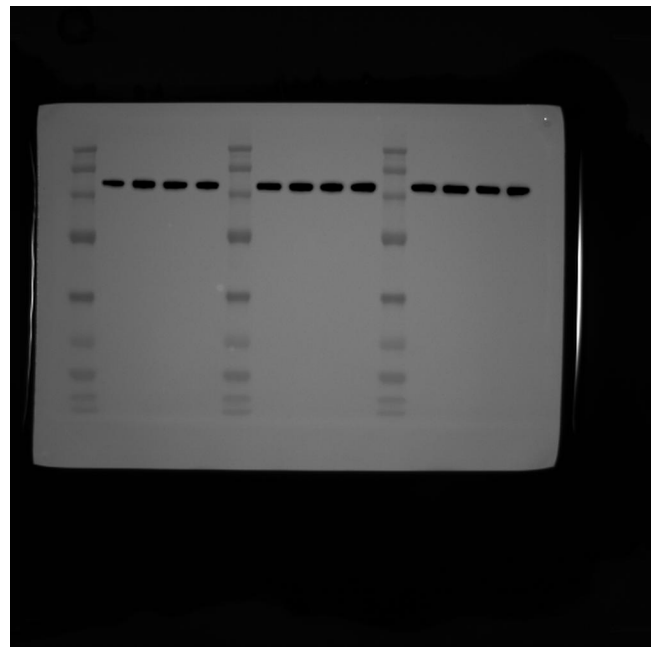

NLRP3

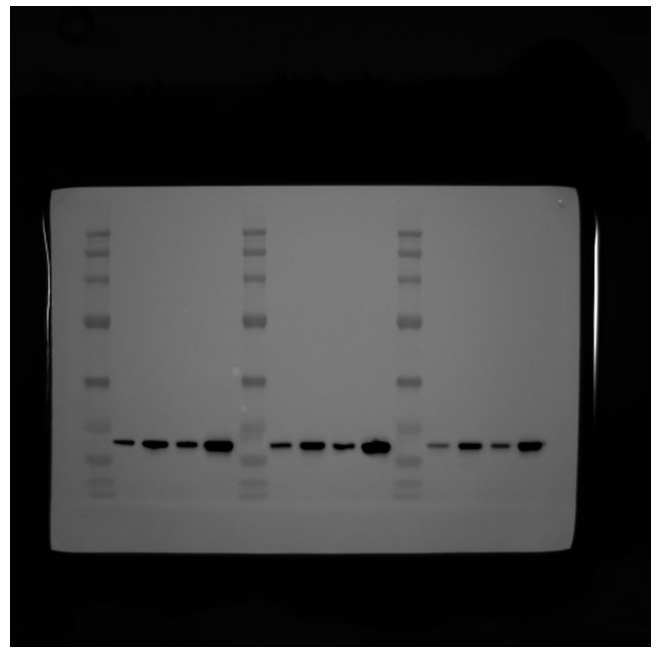

Caspase 1

IP: Caspase 1

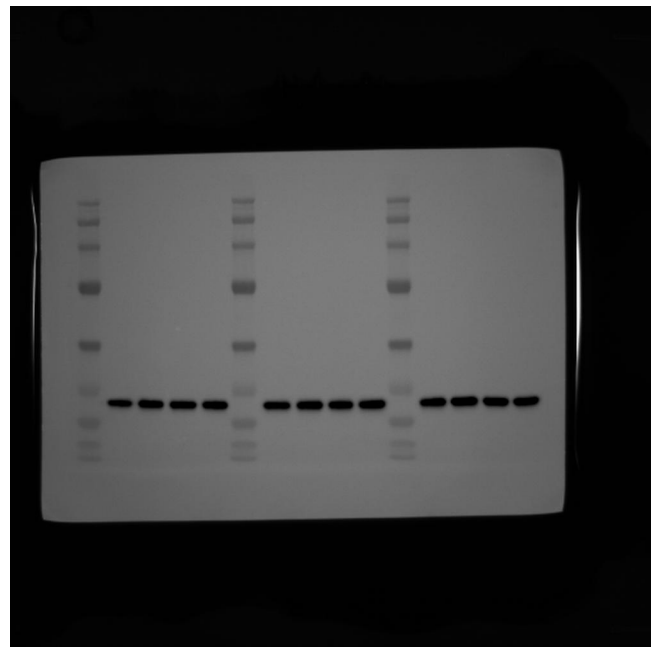

Caspase 1

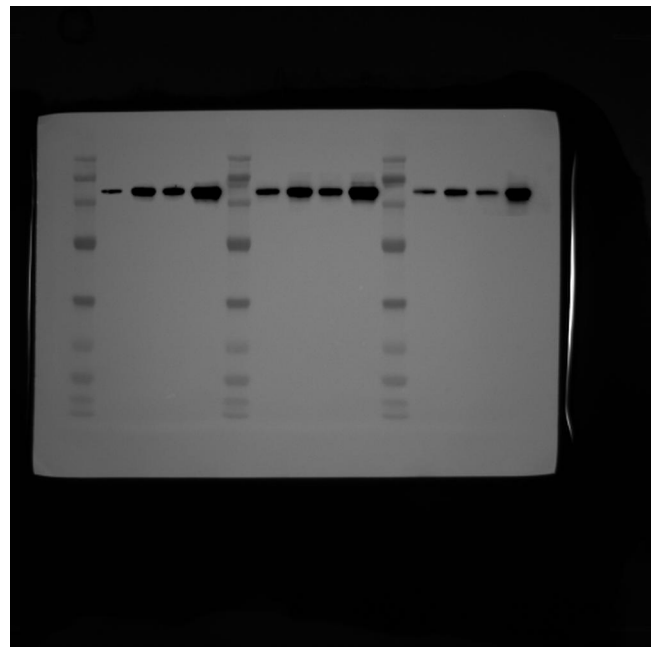

NLRP3

Figure 7A

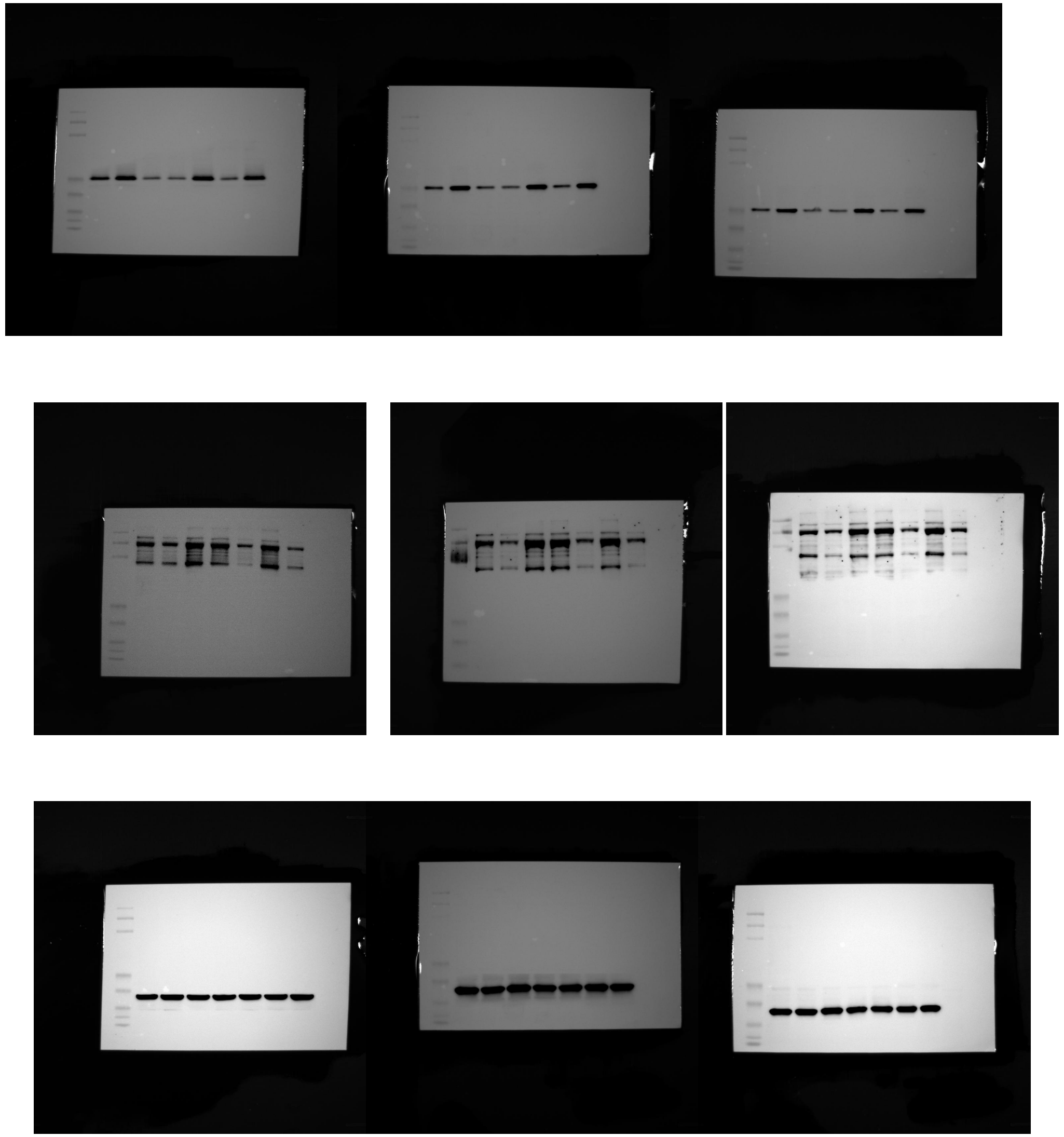

TXNIP

a-Klotho

GAPDH

Figure 8A

Input

NLRP3

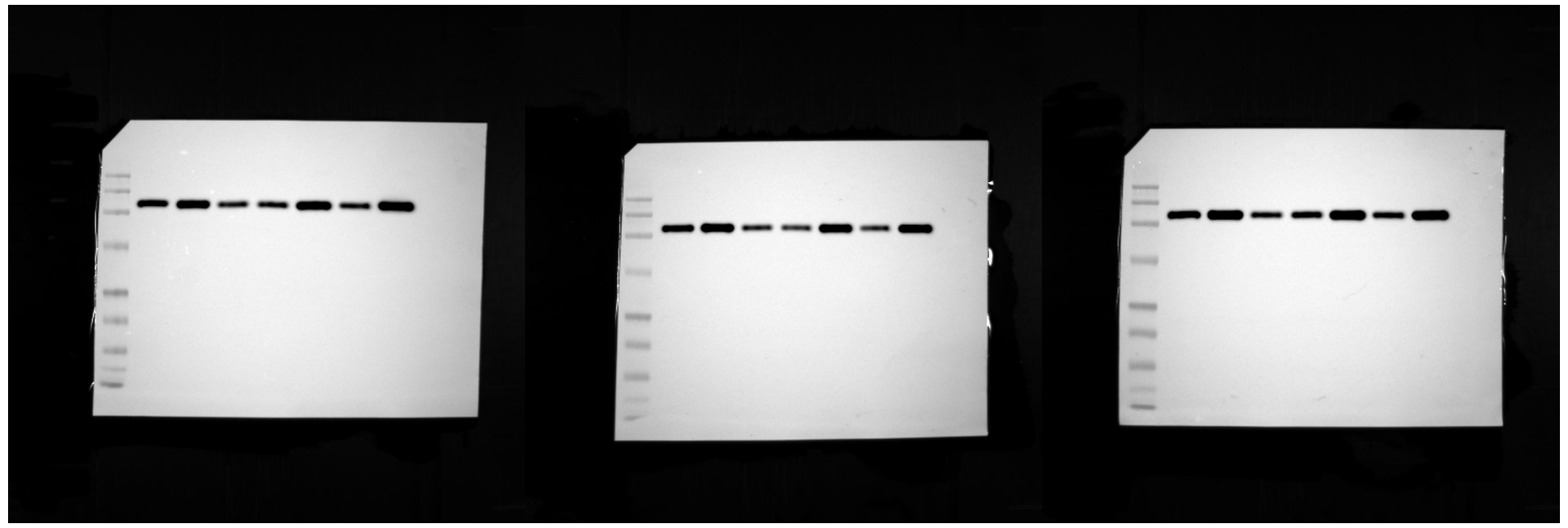

ASC

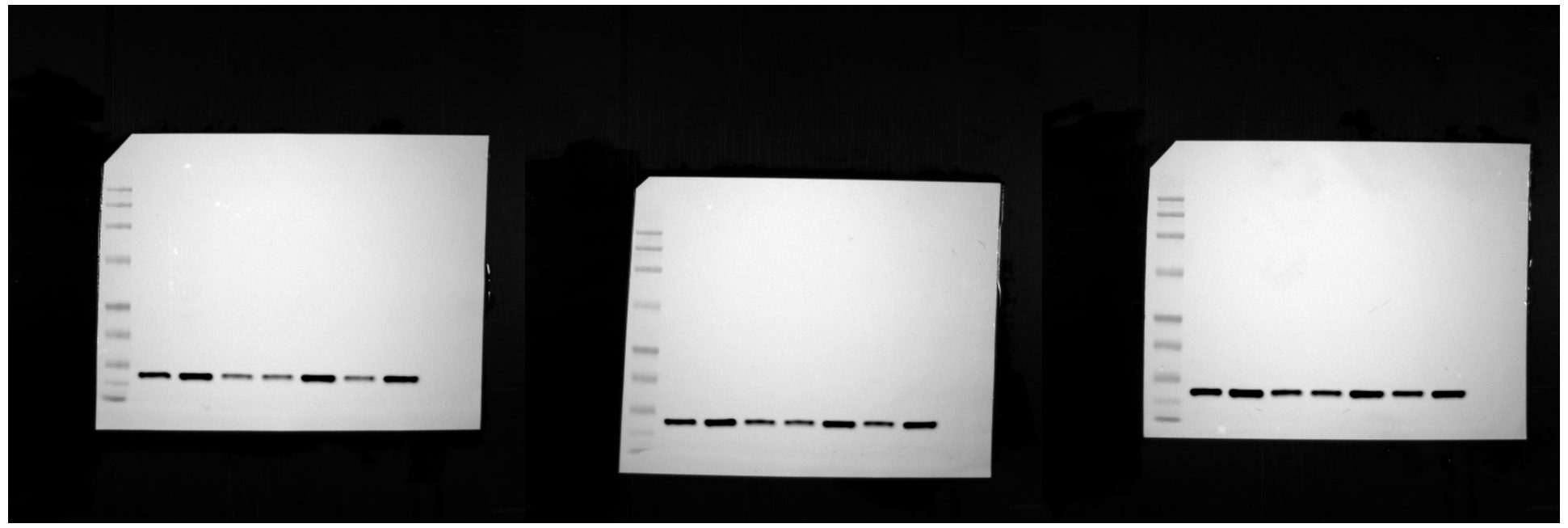

GAPDH

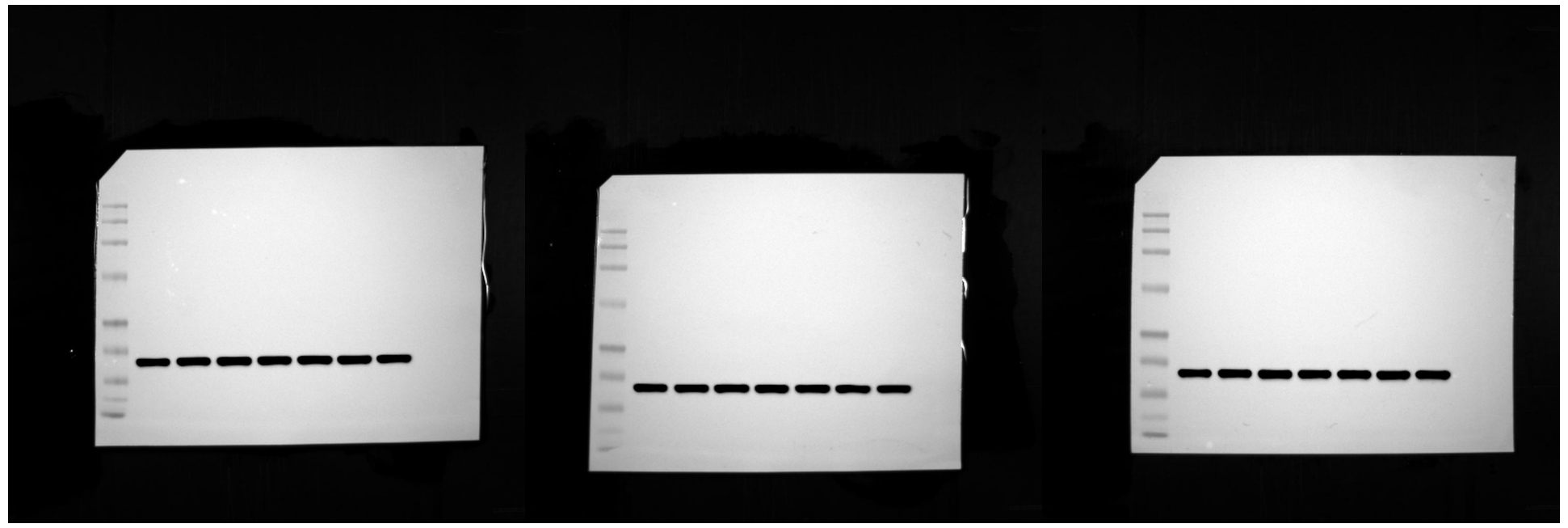

IP: NLRP3

NLRP3

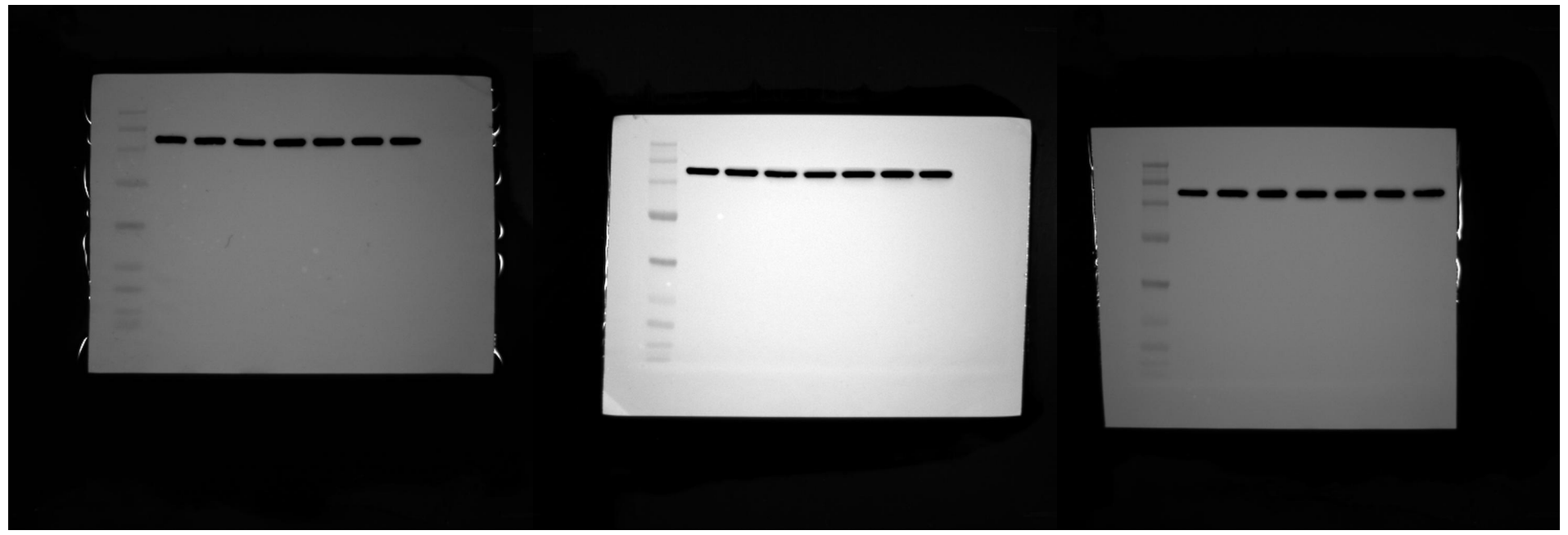

ASC

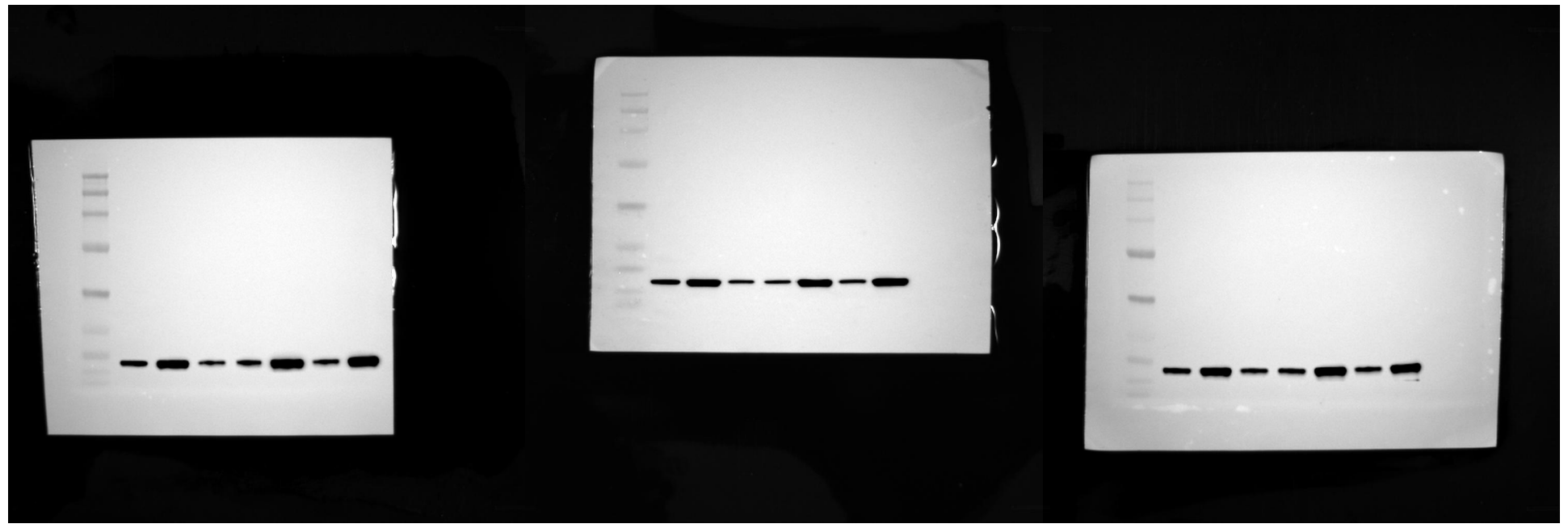

IP: ASC

ASC

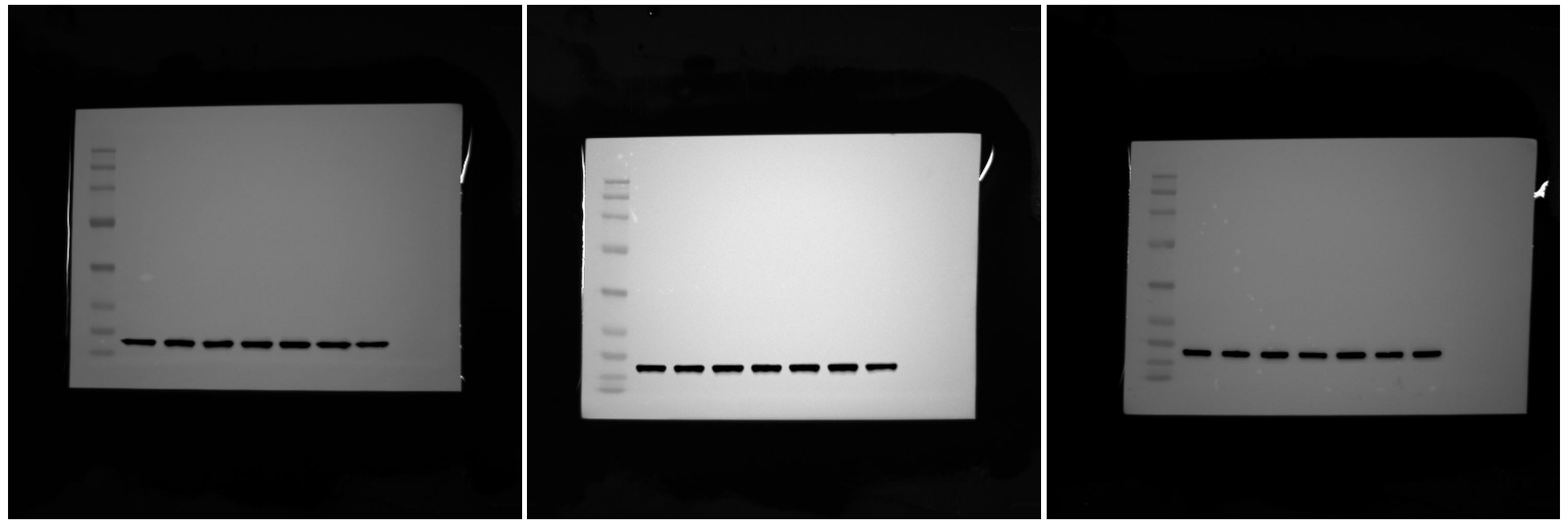

NLRP3

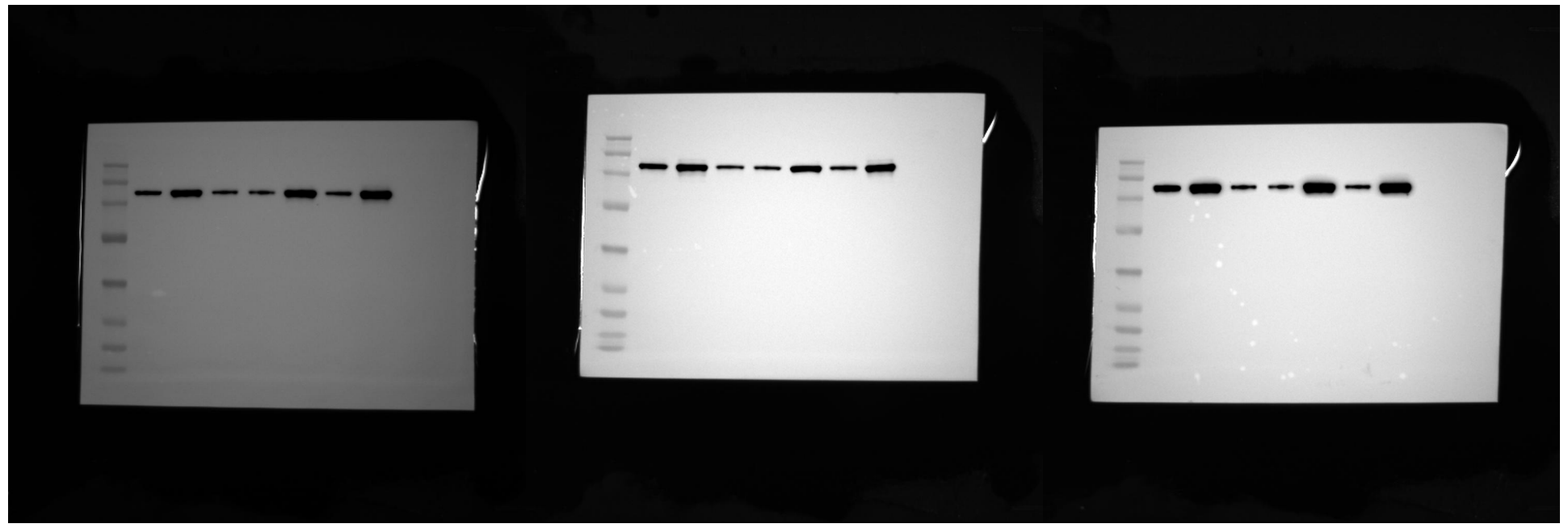

Figure 8D

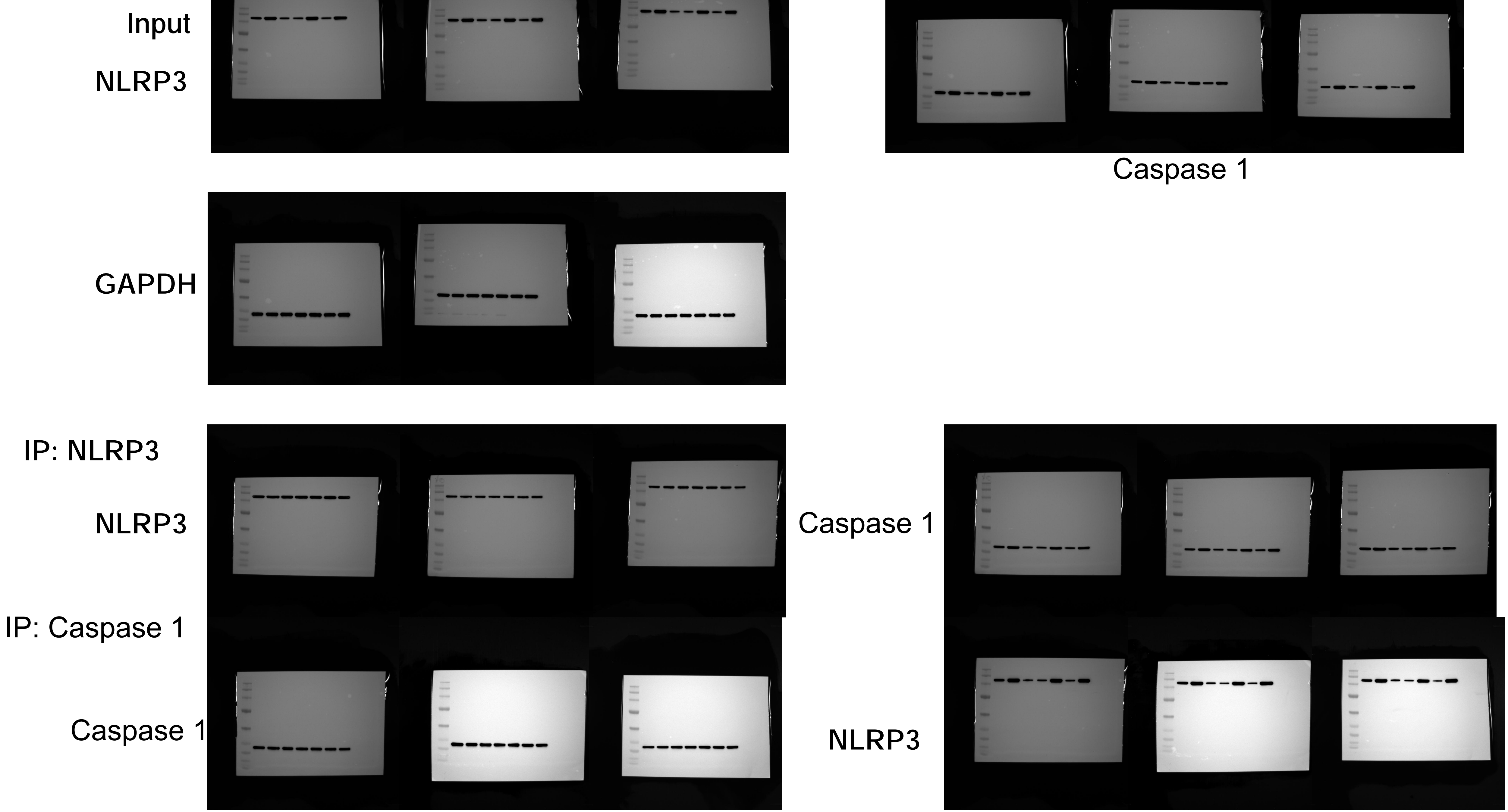
Input
NLRP3
Caspase 1
GAPDH
IP: NLRP3
NLRP3
Caspase 1
IP: Caspase 1
Caspase 1
NLRP3

Supplementary Figure 2A

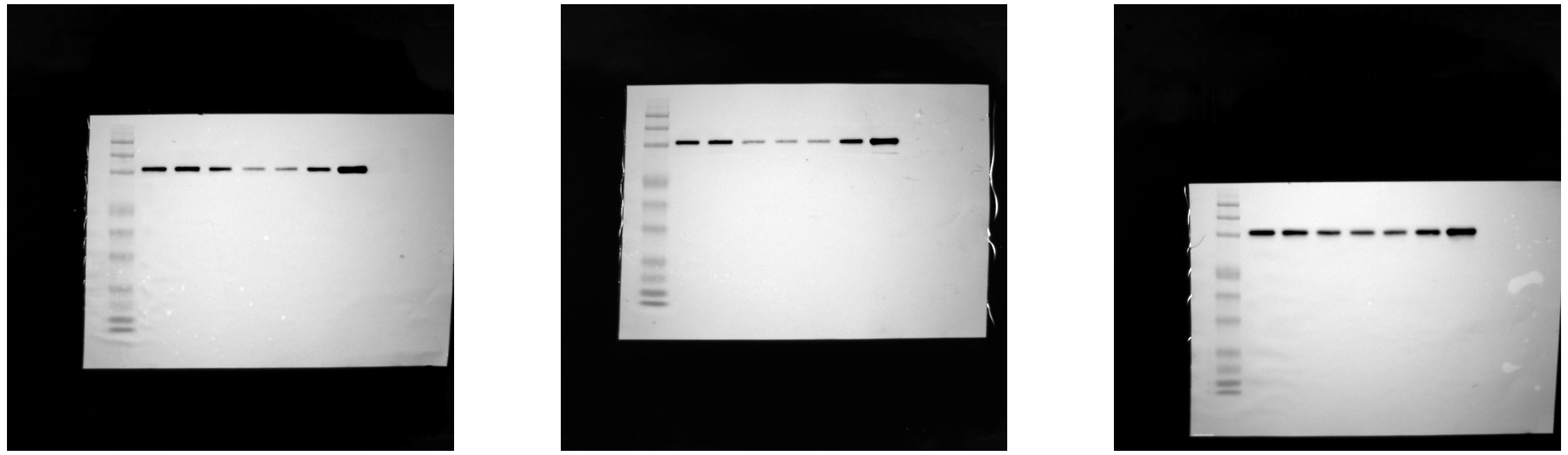

a-Klotho

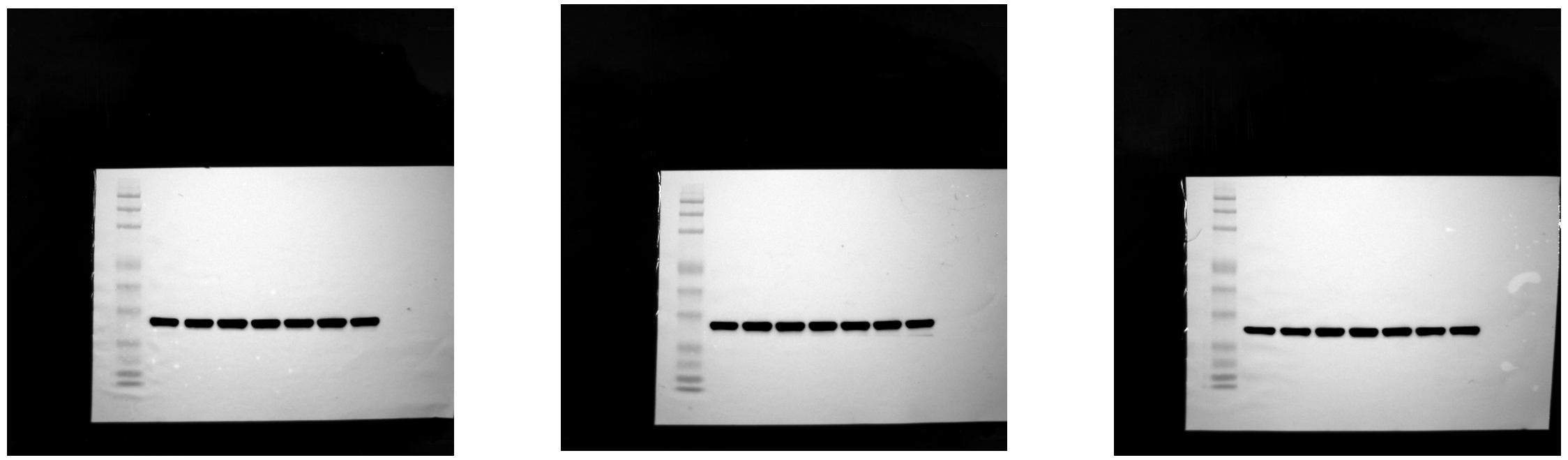

GAPDH

Supplementary Figure 2B

TXNIP

GAPDH

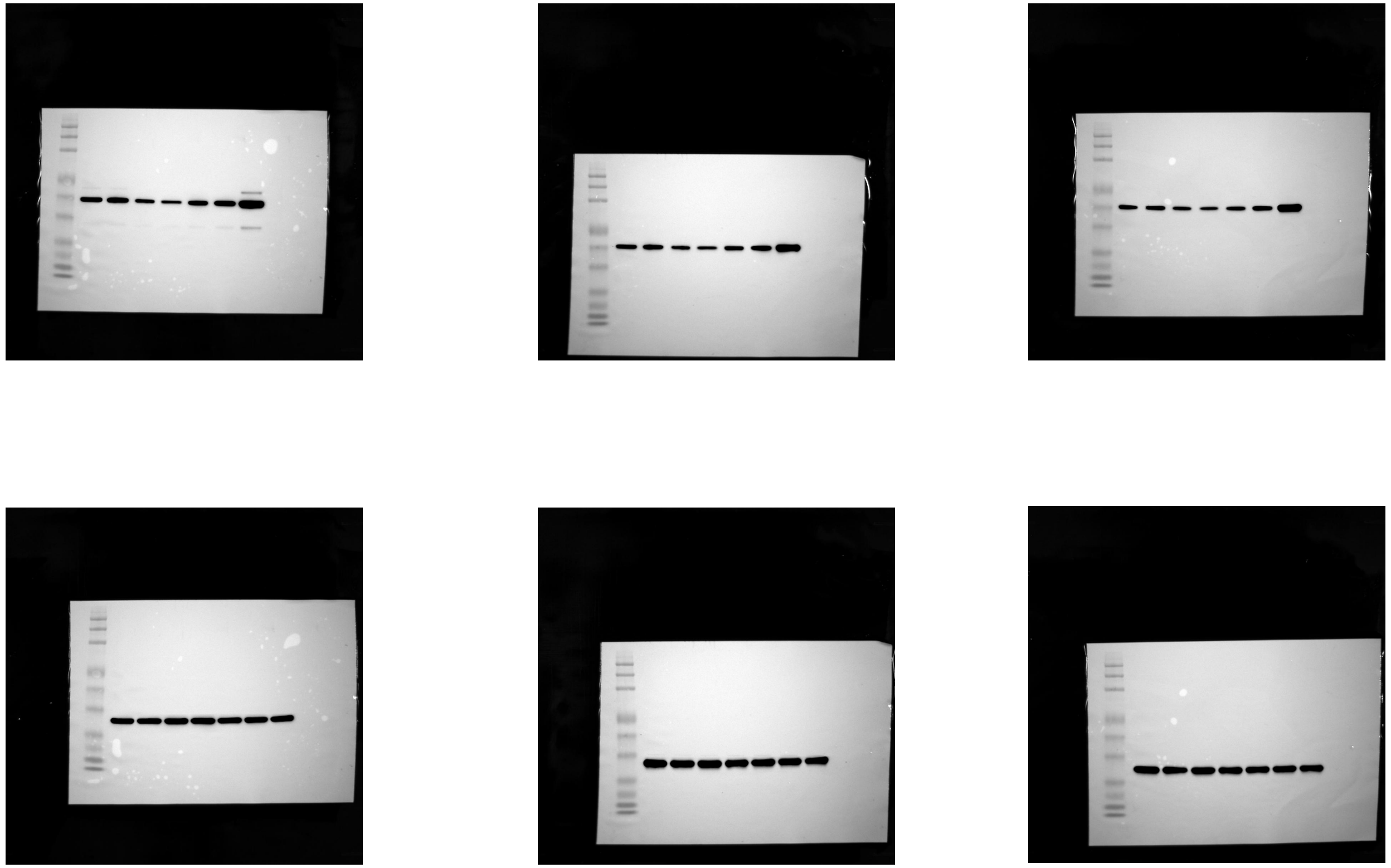

Data S2. Representative unedited microscopy and imaging source images.

Figure 2A

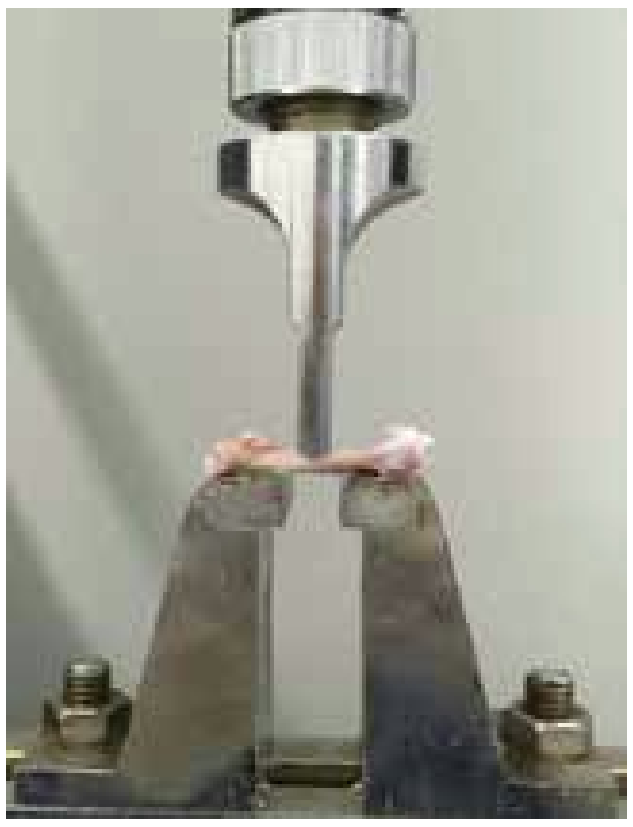

Figure 2G

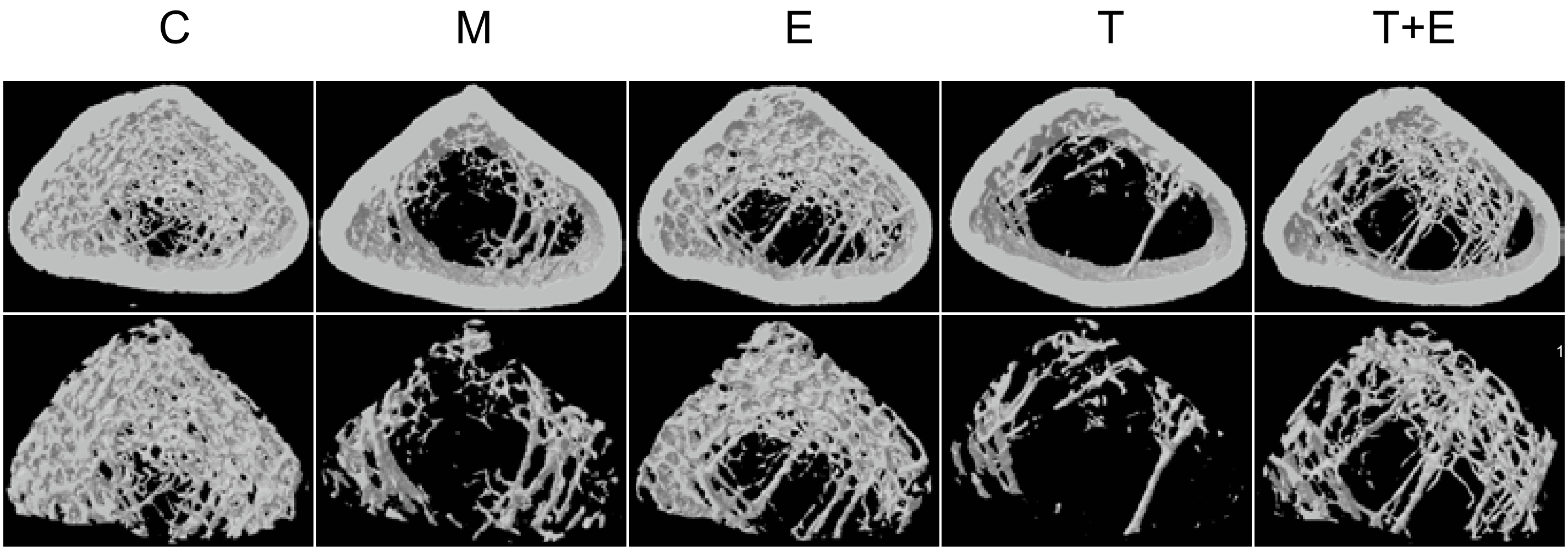

Figure 2L

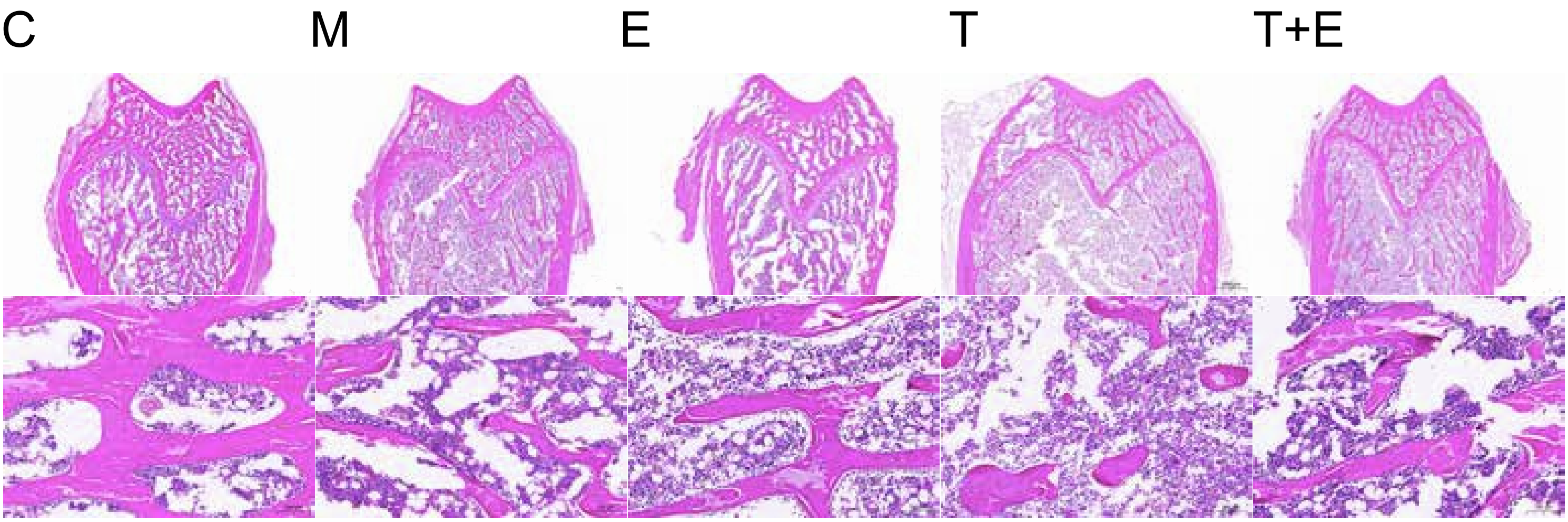

Figure 6G

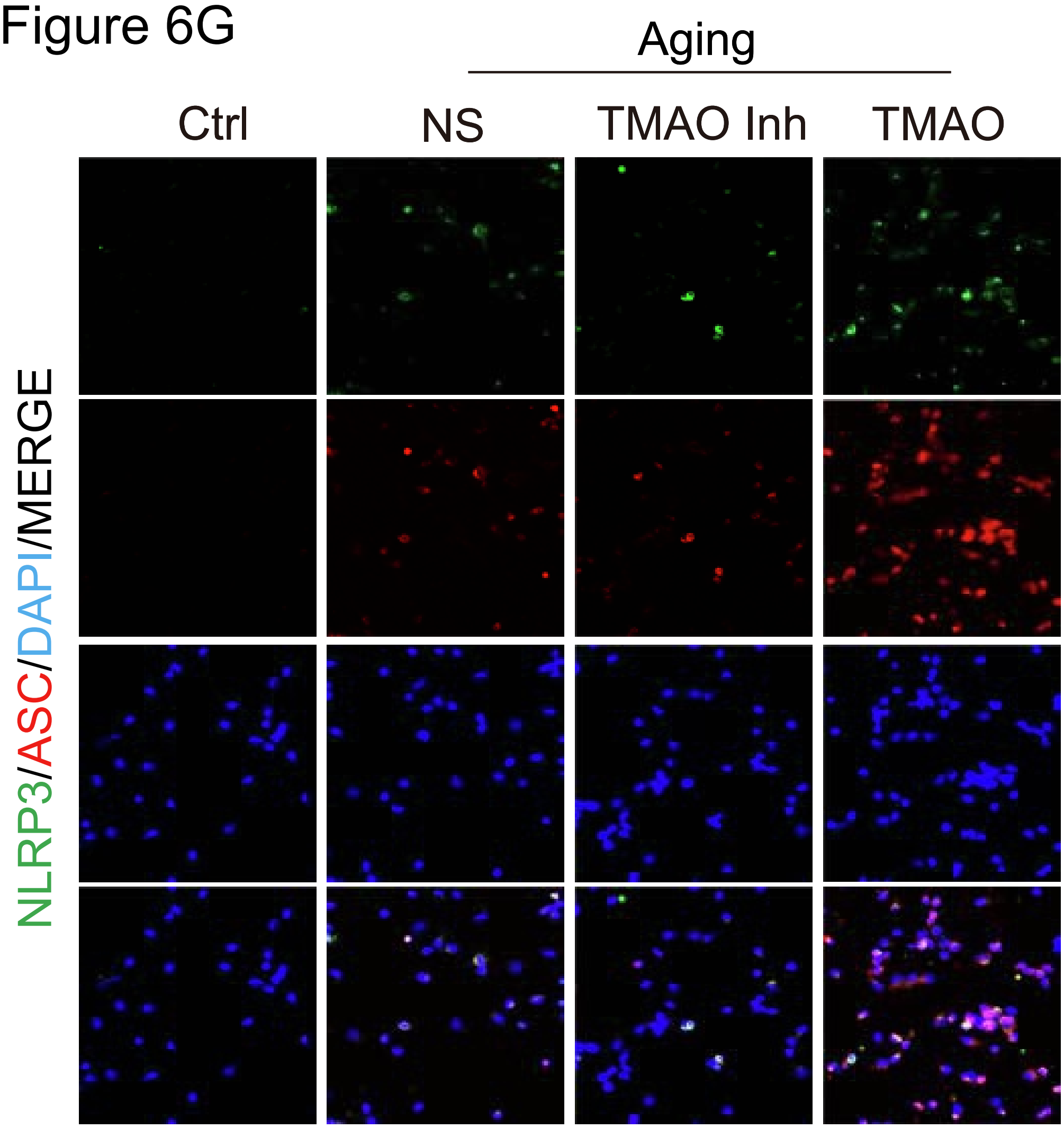

Figure 6H

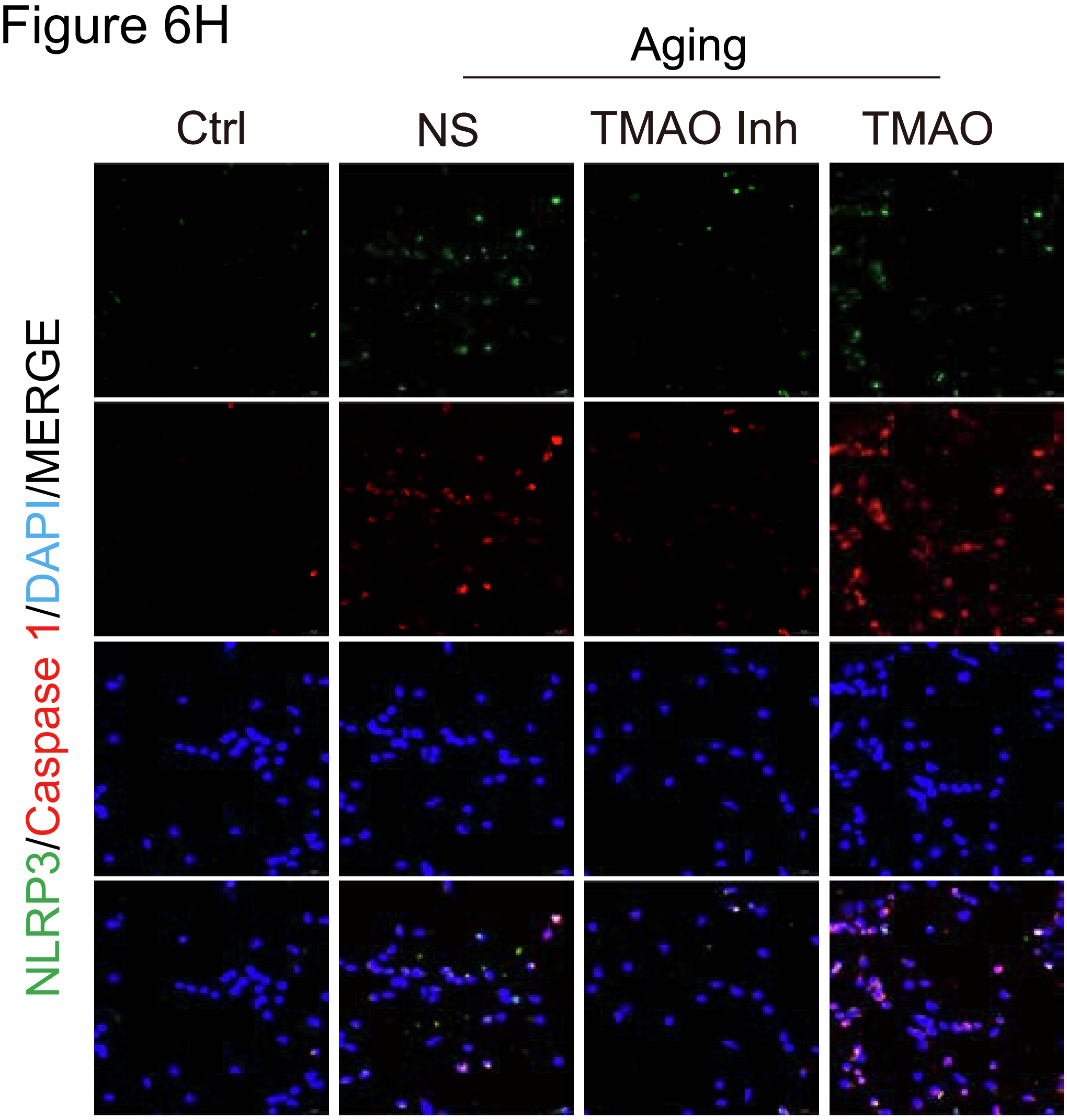

Supplement: Supplementary file 1 [file mmc1.pdf]
